# Supplementary material for: Breast-Specific Epigenetic Regulation of DeltaNp73 and Its Role in DNA-Damage-Response of BRCA1-Mutated Human Mammary Epithelial Cells
Source: Cancers (Basel). 2020 Aug 21;12(9):2367. doi: 10.3390/cancers12092367 (PMC7564633; doi:10.3390/cancers12092367)
Supplement: Supplementary file 1 [file cancers-12-02367-s001.pdf]

Supplementary Information

Breast-specific epigenetic regulation of deltaNp73 and its role in DNA-damage-response of *BRCA1*-mutated human mammary epithelial cells

Ayelet Avraham, Susanna Feldman, Sean Soonweng Cho, Ayala Kol, Lior Heler, Emmanuela Riklin-Nahmias, Avishay Sella, Tamar Karni, Tanir M. Allweis, Saraswati Sukumar, Ella Evron.

Corresponding author: Ayelet Avraham, email: AyeletA@shamir.gov.il

Table of Contents

1. **Supplementary Figures**

1.1 Figure S1. Schematic structure of the *TP73* gene.

1.2 Figure S2 a, b. Cell cytotoxicity in the various human normal primary epithelial cells following treatment with cisplatin and doxorubicin.

1.3 Figure S2 c. Induction of *PUMA*, *p21* and *NOXA* by DNA damage in HMECs and in other types of human epithelial cells.

1.4 Figure S3 a, b The *deltaNp73* promoter is unmethylated in HMECs and fully methylated in white blood cells of *BRCA1* mutation carriers and non-carriers.

1.5 Figure S3 c. Linear mixed model analysis compared the decrease in cell viability in HMEC *BRCA1*-wt and in HMECs *BRCA1*-mut.

1.6 Figure S4 a-c. DNA methylation and induction of the *deltaNp73* and the *TAp73* gene isoforms by DNA damage in MCF10A.

1.7 Figure S4 d. Inhibition of deltaNp73 by siRNA in MCF10A and in MCF10A/ BRCA1-KD cells.

1.8 Figure S4 e. Linear mixed model analysis compared the decrease in cell viability in MCF10A and in MCF10A/BRCA-KD following deltaNp73 inhibition and treatment with cisplatin.

1.9 Figure S4 f, g. Inhibition of deltaNp73 by siRNA caused a decrease in cell viability (XTT) following exposure to doxorubicin in MCF10Aand this effect was augmented in MCF10A/BRCA1-KD.

1.10 Figure S5 a, b. Expression of BRCA1 in MCF10A clones stably transfected with shBRCA #1 and #2 and flag-iΔNp73. Comparison of cell viability curves in response to cisplatin between these clones.

1.11 Figure S6a, b. Expression of BRCA1 and deltaNp73 mRNA and protein in HMECs in response to cisplatin.

1.12 Figure S7. DNA methylation across the entire *TP73* gene among breast cancer subtypes - TCGA analysis.

1.13 Figure S8. Western blots showing co-immunoprecipitation experiments of deltaNp73 with BRCA1 and 53BP1.

1.14 Full images of all western blots without processing.

2. **Supplementary Methods**

2.1 Quantitative Methylation-Specific PCR (Q-MSP)

2.2 Illumina methylation array (methyl27K) probes at the TAp73 and deltaNp73 promoters

2.3 Primers used for Q-MSP and RT-Qpcr

2.4 si/shRNA Sequences used for deltaNp73and BRCA1 knockdown and control.

1.1 Figure S1

Adapted from:

Killick R, Niklison-Chirou M, Tomasini R, Bano D, Rufini A, Grespi F, Velletri T, Tucci P, Sayan BS, Conforti F, Gallagher E, Nicotera P, Mak TW, Melino G, Knight RA, Agostini M. p73: a multifunctional protein in neurobiology.

Mol Neurobiol. 2011 Apr;43(2):139-46. doi: 10.1007/s12035-011-8172-6.

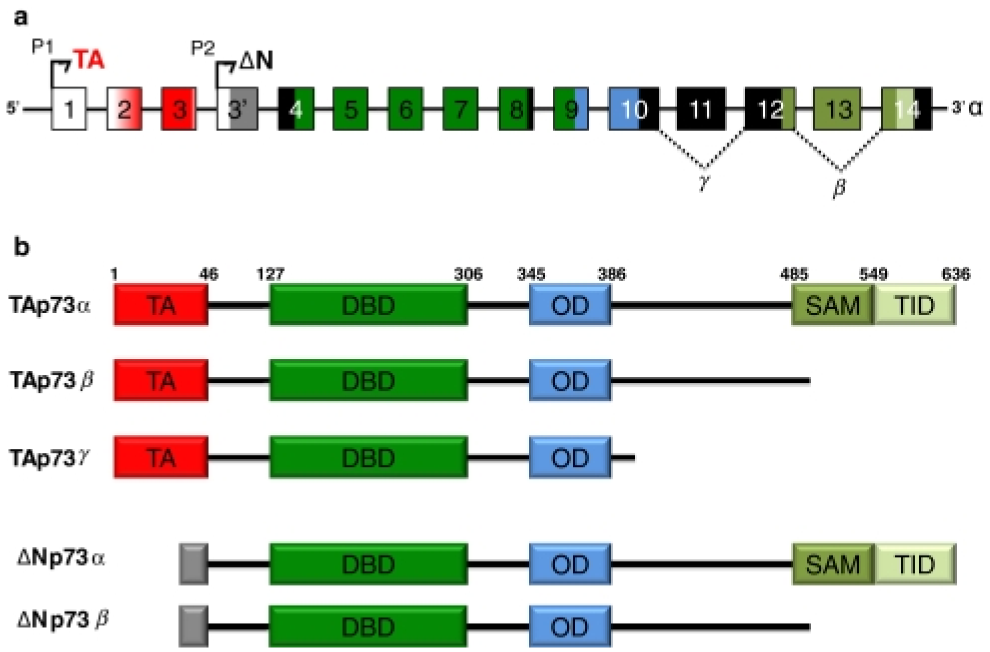

Fig. S1 Schematic structure of the *TP73* gene.

a. Genomic organization of the *TP73* gene: exons, introns and alternative splicing sites. The colors represent the specific protein domains shown below. The P1 promoter generates the TA isoforms, while the P2 promoter generates the ΔN isoforms. **b** Schematic representation of the domains encoded by the different isoforms of *p73*. TA - transactivation domain, DBD - DNA-binding domain, OD – oligomerization domain, SAM – sterile alpha motif domain, TID transactivation inhibitory domain.

1.2 Figure S2

a

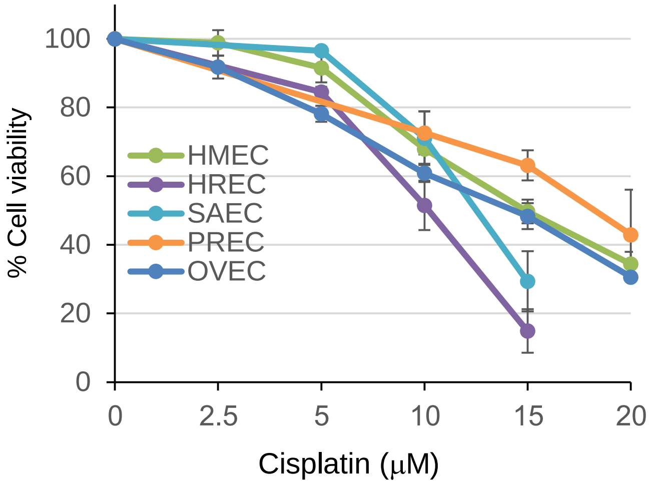

Expression of ΔNp73 (RU)		
**Cisplatin (μM)**	**0**	**15**
HMEC	4.2	12.3
SAEC	0.7	3.0
PREC	0.6	4.7
HREC	0.7	0.4
OVEC	0.3	0.8

b

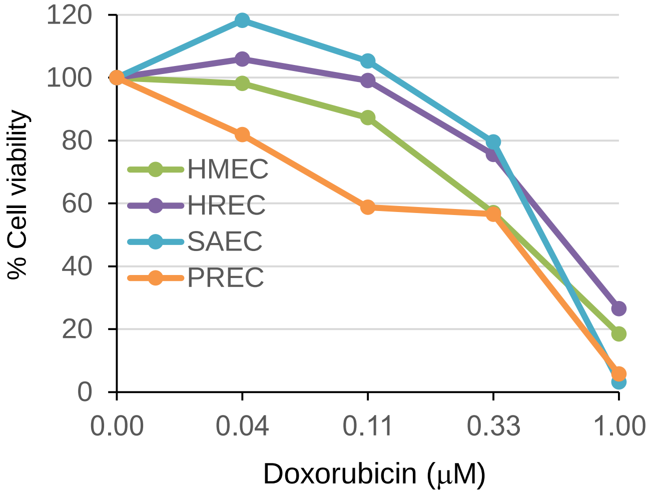

Expression of ΔNp73 (RU)			
**Doxorubicin (μM)**	**0**	**0.33**	**1**
HMEC	2.78	52.85	74.14
SAEC	0.16	39.61	1.38
PREC	0.04	9.33	28.7
HREC	0.00	0.03	0.41

Fig. S2 Cell cytotoxicity in the various human normal primary epithelial cells following treatment with cisplatin (a) and doxorubicin (b). The cells were exposed to increasing doses of cisplatin or doxorubicin for 24 hours and cell viability was measured by XTT. HMECs – mammary, HREC – renal, SAEC – small airways, PREC – prostate, OVEC – ovary. The data represent average of 3 replicates in cells originated from 3 individuals (cisplatin) or 1 individual (doxorubicin). Error bars represent standard error of the mean (S.E.M). The tables on the right indicate that deltaNp73 induction by cisplatin or doxorubicin differed between cell types and these differences did not correlate with cell cytotoxicity.

1.3 Figure S2 c

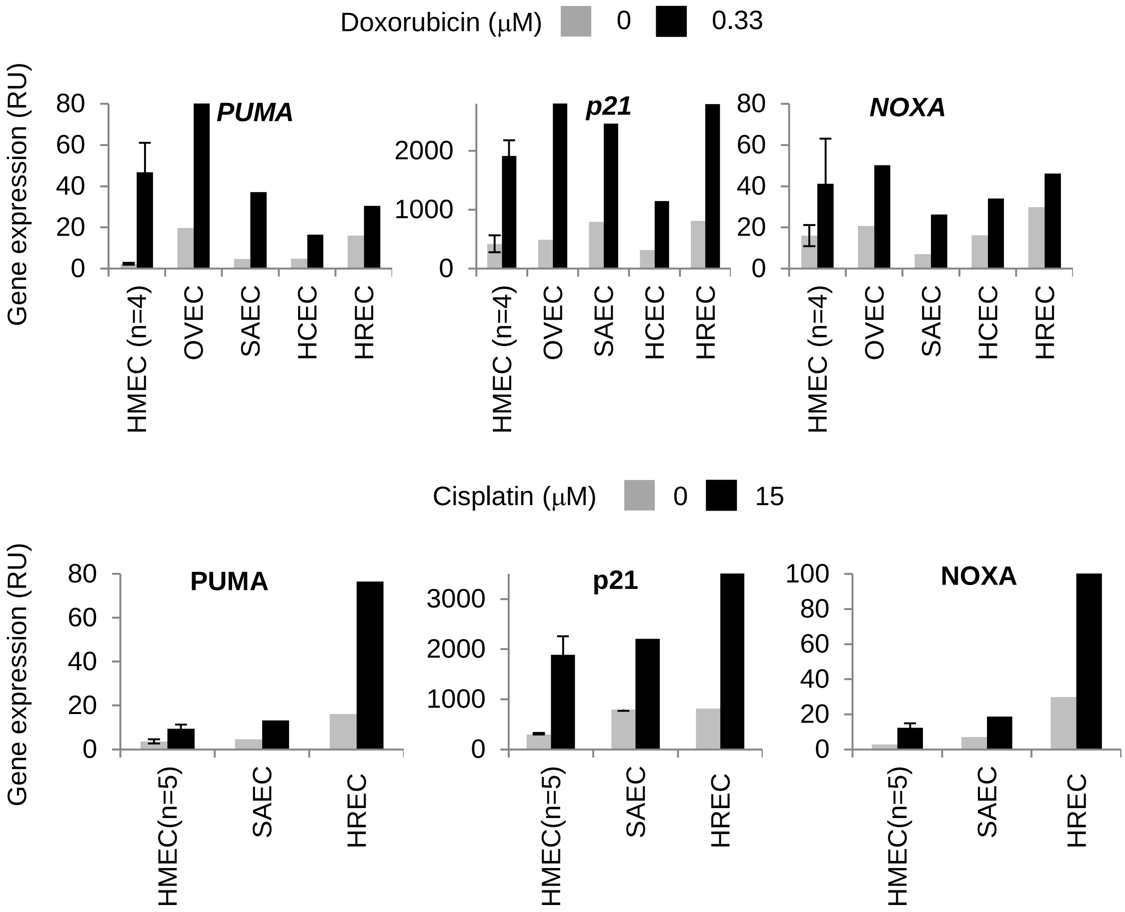

Fig. S2c. Induction of PUMA, p21 and NOXA by DNA damage in HMECs and in other types of human epithelial cells. The cells were exposed to doxorubicin or cisplatin for 24 hours. Gene expression (mRNA) was measured by RT-qPCR and normalized to *GAPDH* (RU = $2^{\Delta CT}$ x 10^4). Epithelial cells tested: HMECs – mammary, SAEC – small airways, HREC – renal, PREC – prostate, OVEC – ovary, HCEC – colon. Error bars represent standard error of the mean (S.E.M). Number of individual tissues (n) is marked when n>1.

1.4 Figure S3 a

a

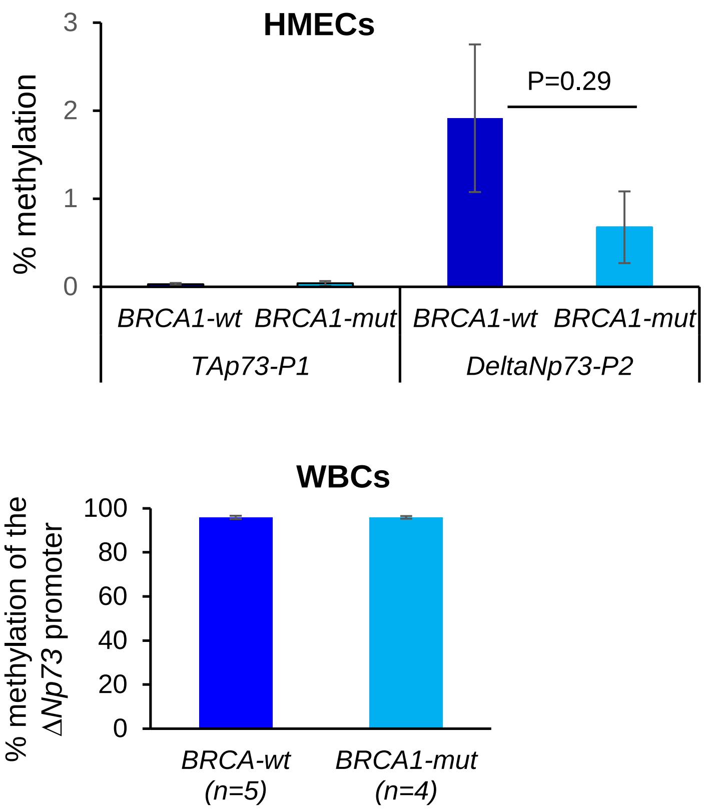

Fig. S3a The *deltaNp73* promoter is unmethylated in HMECs and fully methylated in white blood cells of *BRCA1* mutation carriers and non-carriers. Upper panel: DNA methylation of the *deltaNp73* promoter in primary human mammary epithelial cells (HMECs) from *BRCA1* mutation carriers (*BRCA1*-mut, n=8) and non-carriers (*BRCA1*-wt, n=14). **Lower panel:** DNA methylation of the *deltaNp73* promoter in white blood cells (WBCs) from *BRCA1* mutation carriers (*BRCA1*-mut, n=4) and non-carriers (*BRCA1*-wt, n=5). Methylation was measured by quantitative methylation specific PCR (qMSP). T-test showed no significant difference between the groups.

Note: The Y-axis scale is different in each panel.

1.4 Figure S3 b

b

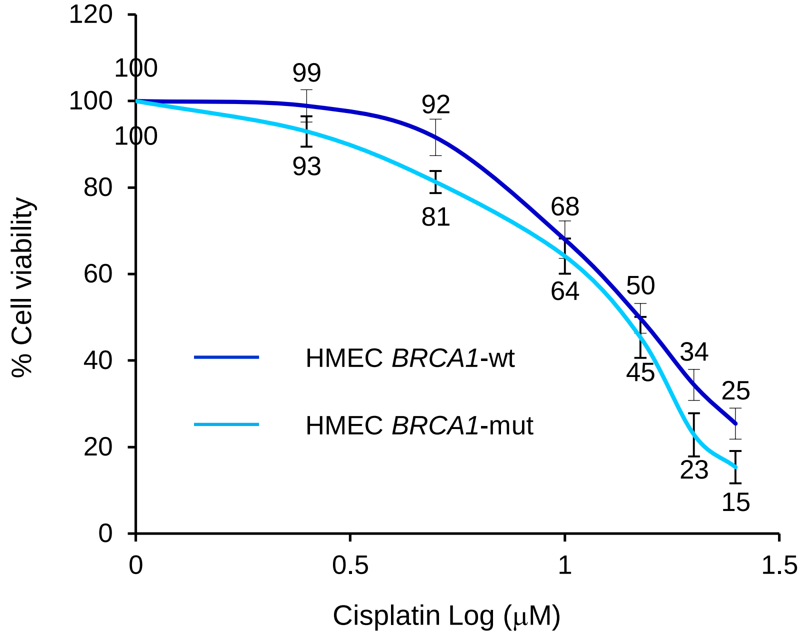

Fig. S3b Cell viability in response to DNA damage in *BRCA1*-wt and *BRCA1*-mut HMECs. Dose response curves of *BRCA1*-wt (n=7 individuals) and *BRCA*-mut (n=5 individuals) HMECs show that the *BRCA1*-mut cells were more sensitive to cisplatin than the *BRCA1*-wt. The data are similar to those presented in Fig.3 b-e (siControl group). The experiment was repeated twice for each individual, and XTT was done in triplicates. Error bars represent S.E.M.

1.5 Figure S3 c

c

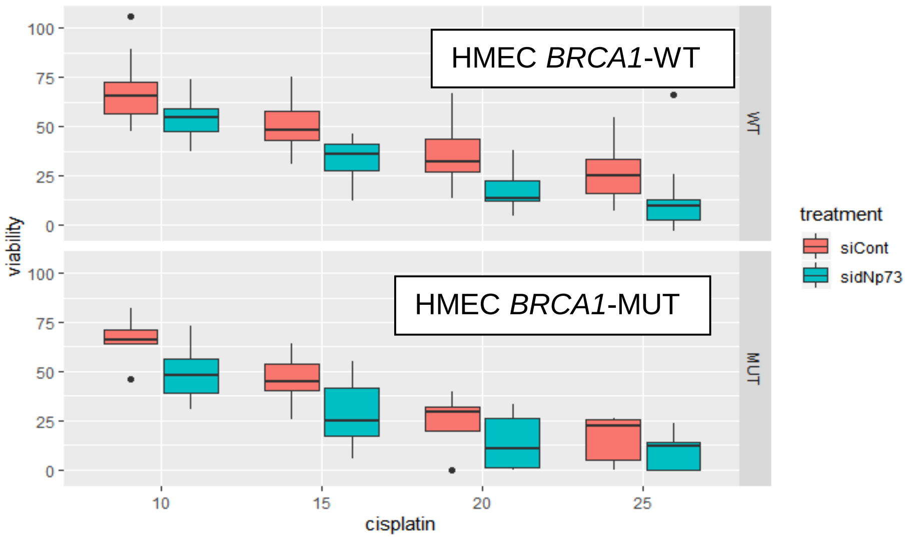

siCont vs sidNp73	Estimate	Error	df	t value	Pr(>|t|)	
**HMEC BRCA1/wt**	15.4426-	5.75597	103.0342	2.683-	**0.0085**	**
**HMEC BRCA1/mut**	23.9086-	6.412	62.0284	3.729-	**0.000419**	***

Fig. S3 c Linear mixed model analysis compared the decrease in cell viability in HMECs *BRCA1*-wt (n=11, upper panel) **and in HMECs *BRCA1*-mut** (n=9, lower panel), between cells transfected with siRNA control (orange) to siΔNp73 (green), following treatment with 10-25 μM cisplatin. Each box displays lower Q1 and upper Q3 with a black line representing the mean, and S.E.M error bars. The table below shows analysis parameters and p-values for each comparison. The data for the siΔNp73 combines the two different siΔNp73 sequences.

1.6 Figure S4 a-c

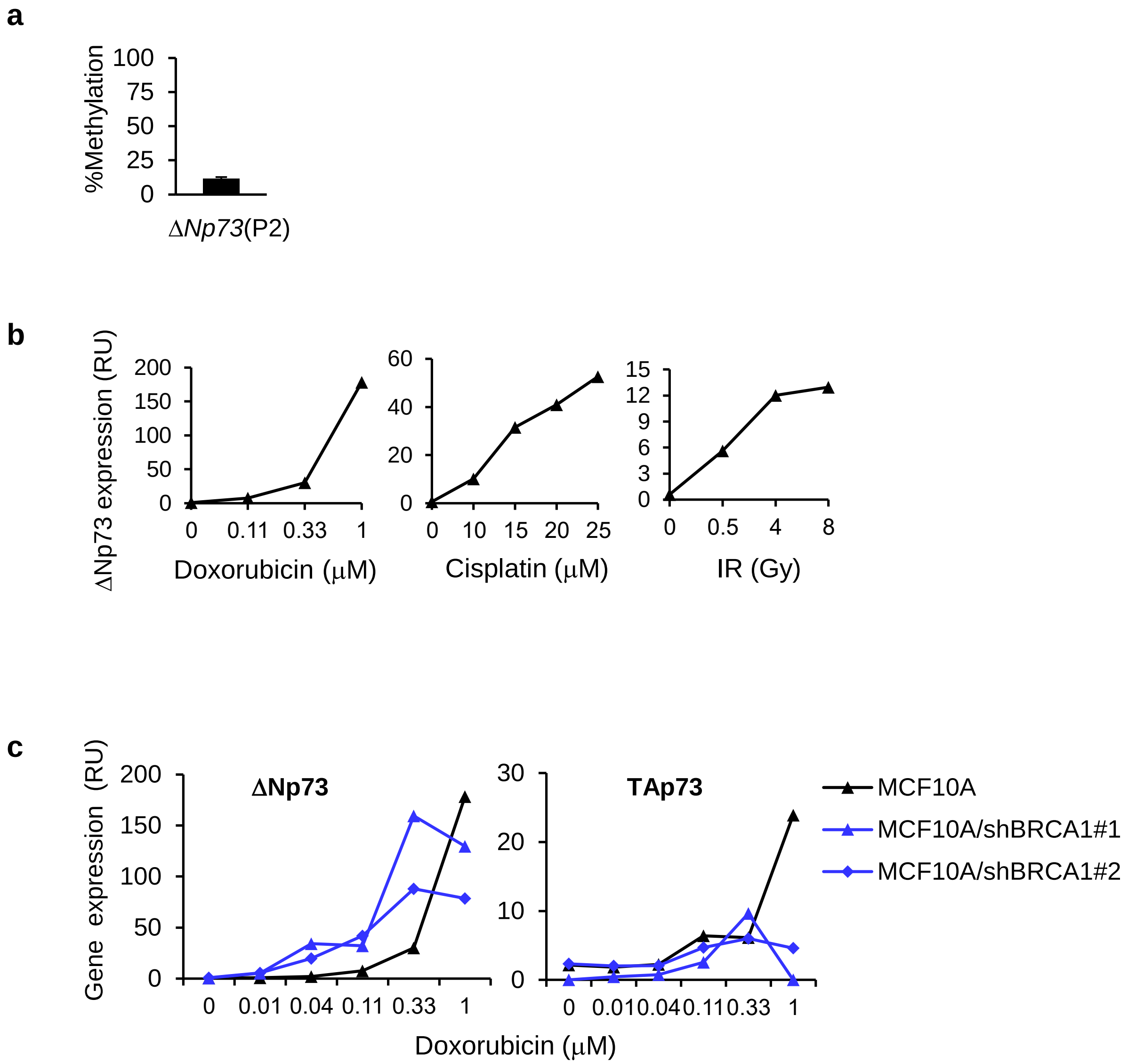

Fig. S4 DNA methylation and induction of the *deltaNp73* and the *TAp73* gene isoforms by DNA damage in MCF10A. a Methylation at the *delatNp73* promoter (P2) in MCF10A (average of 12 independent Q-MSP measures). **b** Induction of *deltaNp73* in MCF10A by various DNA damage stimuli for 24 hours. **c** Induction of *deltaNp73* and *TAp73* by doxorubicin for 24 hours in MCF10A and in BRCA1-KD MCF10A. Gene expression (mRNA) was measured by RT-qPCR and normalized to GAPDH (RU = $2^{\Delta CT} \times 10^4$).

1.7 Figure S4 d

d

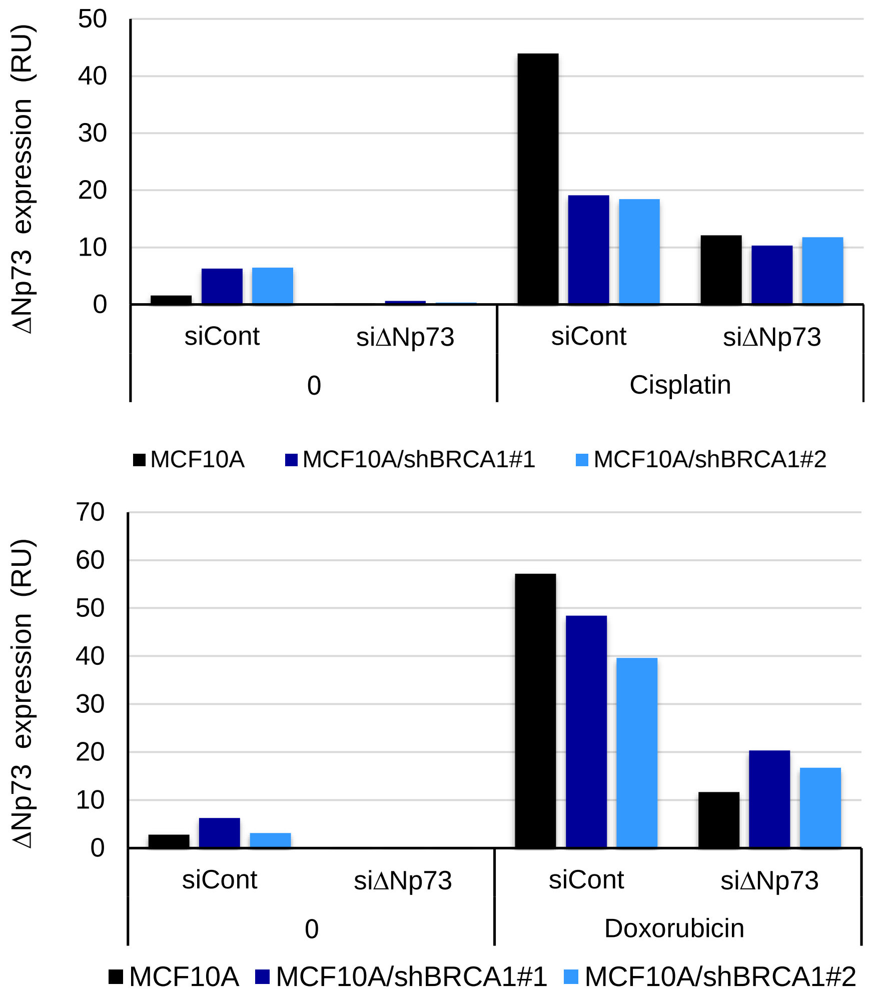

Fig S4 d. Inhibition of deltaNp73 by siRNA in MCF10A and in MCF10A/ BRCA1-KD cells. The cells were untreated (0) or treated with 15 μM cisplatinum or 0.11 μM doxorubicin for 24 hours. Gene expression (mRNA) was measured by RT-qPCR and normalized to GAPDH (RU = $2^{\Delta CT}$ x 10^4).

1.8 Figure S4e

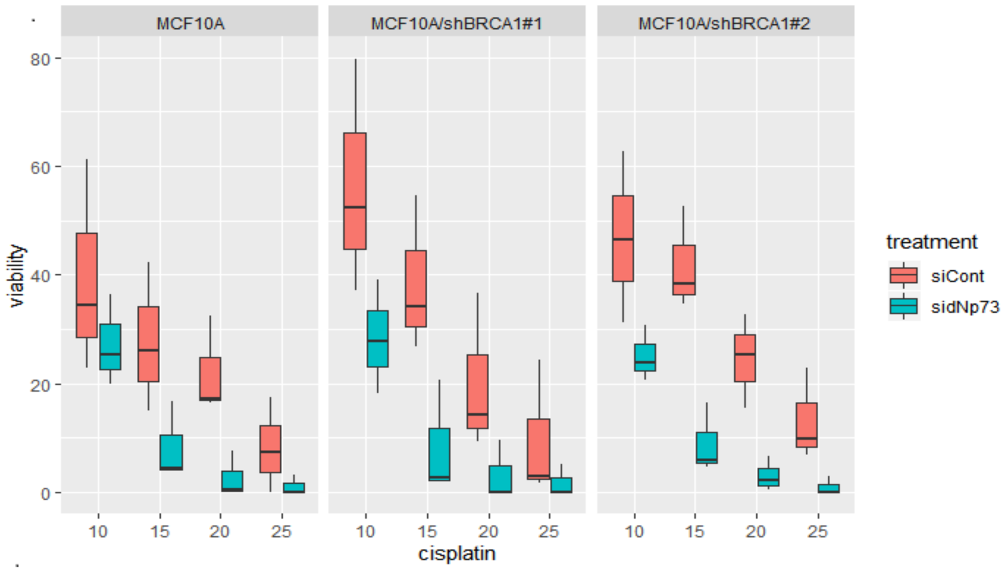

siCont vs sidNp73	Estimate	Std. Error	df	t value	Pr(>|t|)	
MCF10A	19.87-	8.87	18	2.2-	**0.0379**	*
MCF10A/shBRCA1#1	46.62-	9.18	18	5.1-	**7.80E-05**	***
MCF10A/shBRCA1#2	36.00-	7.49	18	4.8-	**1.41E-04**	***

Fig. S4 e Linear mixed model analysis compared the decrease in cell viability in MCF10A and in MCF10A/BRCAKD following deltaNp73 inhibition and treatment with cisplatin. Analysis compared cell viability in response to increasing concentrations (10-25 μM) of cisplatin, between MCF10A cells that were transfected with non-target siRNA (siControl, orange) and cells transfected with siRNA targeting deltaNp73 (siΔNp73, green). Analysis compared MCF10A and MCF10A harboring shRNA for BRCA1 (shBRCA1#1, #2). Each transfection experiment was repeated 3 times and cytotoxicity measurements were done in triplicates. Each box displays lower Q1 and upper Q3 with a black line representing the mean, and S.E.M error bars. The table below shows the analysis parameters and p-value. The data for the siΔNp73 represent the average of two different siRNA sequences.

1.9 Figure S4 f-g

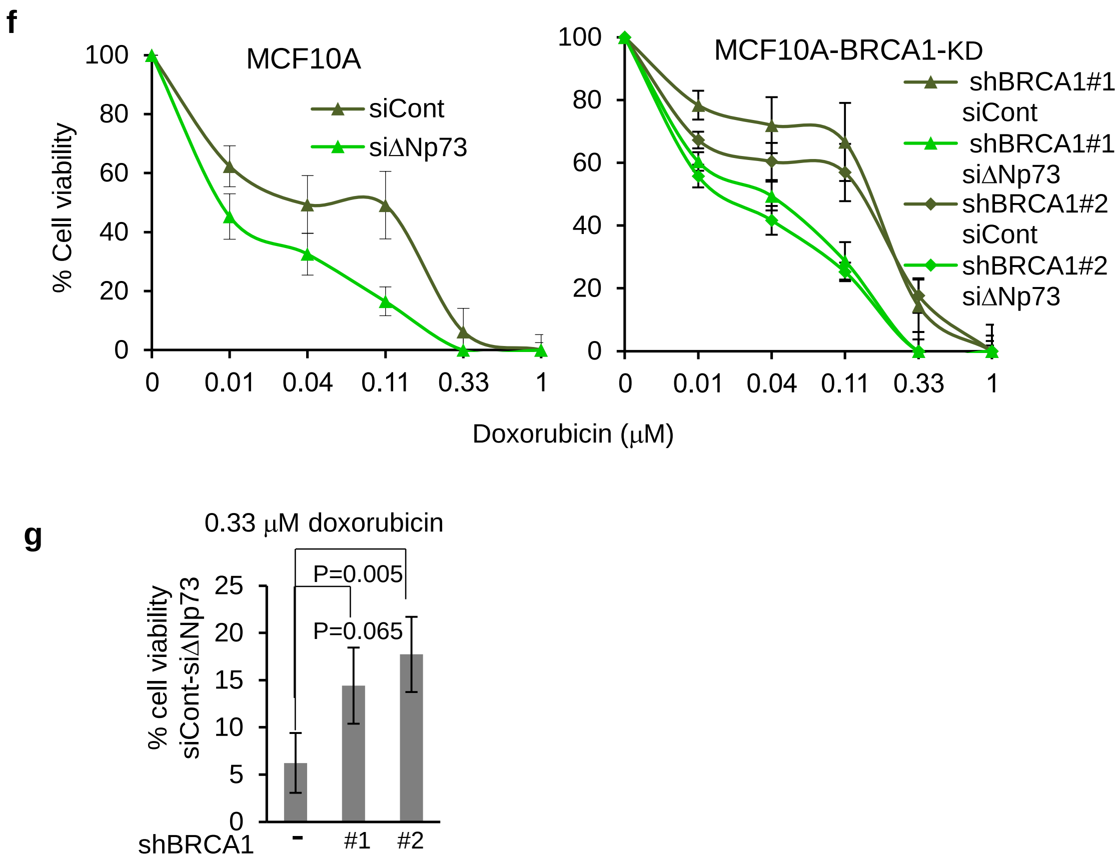

Fig S4 f. Inhibition of deltaNp73 by siRNA caused a decrease in cell viability (XTT) following exposure to doxorubicin in MCF10A (left) and this effect was augmented in MCF10A/BRCA1-KD (right). **g** The difference in cell viability between siControl and siΔNp73 was compared between MCF10A/BRCA1-KD cells and the parental MCF10A cells for data point of 0.33 μM doxorubicin. P-values were calculated by paired student t-test. Gene expression (mRNA) was measured by RT-qPCR and normalized to GAPDH (RU = $2^{\Delta CT}$ x 10^4). Error bars represent S.E.M of 3 independent experiments.

1. 10 Figure. S5 a-b

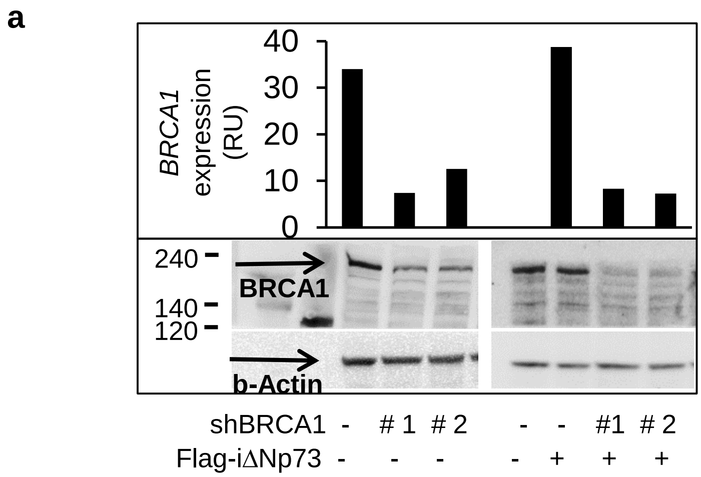

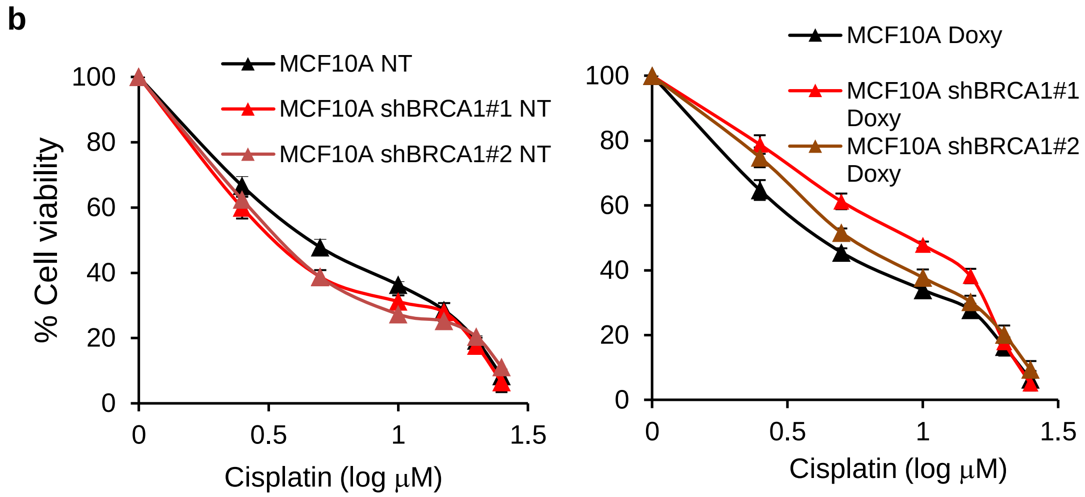

Fig. S5 a Expression of BRCA1 in MCF10A stably transfected with shBRCA and/or stably transfected with flag-iΔNp73. BRCA1 mRNA is shown in upper panel and BRCA1 protein in lower panel. Gene expression (mRNA) was measured by RT-qPCR and normalized to *GAPDH* (RU = $2^{\Delta CT}$ x 10^4). **b** MCF10A BRCA1-KD clones were more sensitive to cisplatin than the parental MCF10A when the cells were not treated (NT) with doxycycline (left). Following induction of deltaNp73 with doxycycline (Doxy, right), the MCF10A BRCA1-KD clones became more resistant to cisplatin than the parental MCF10A. The cells were exposed to increasing doses of cisplatin for 48 hours and viability was measured by XTT. Data represent the mean +/- S.E.M of three independent experiments. Each data point was done in triplicates. These graphs are alternative presentation of the data shown in figure 5.

1.11 Figure S6 a-b

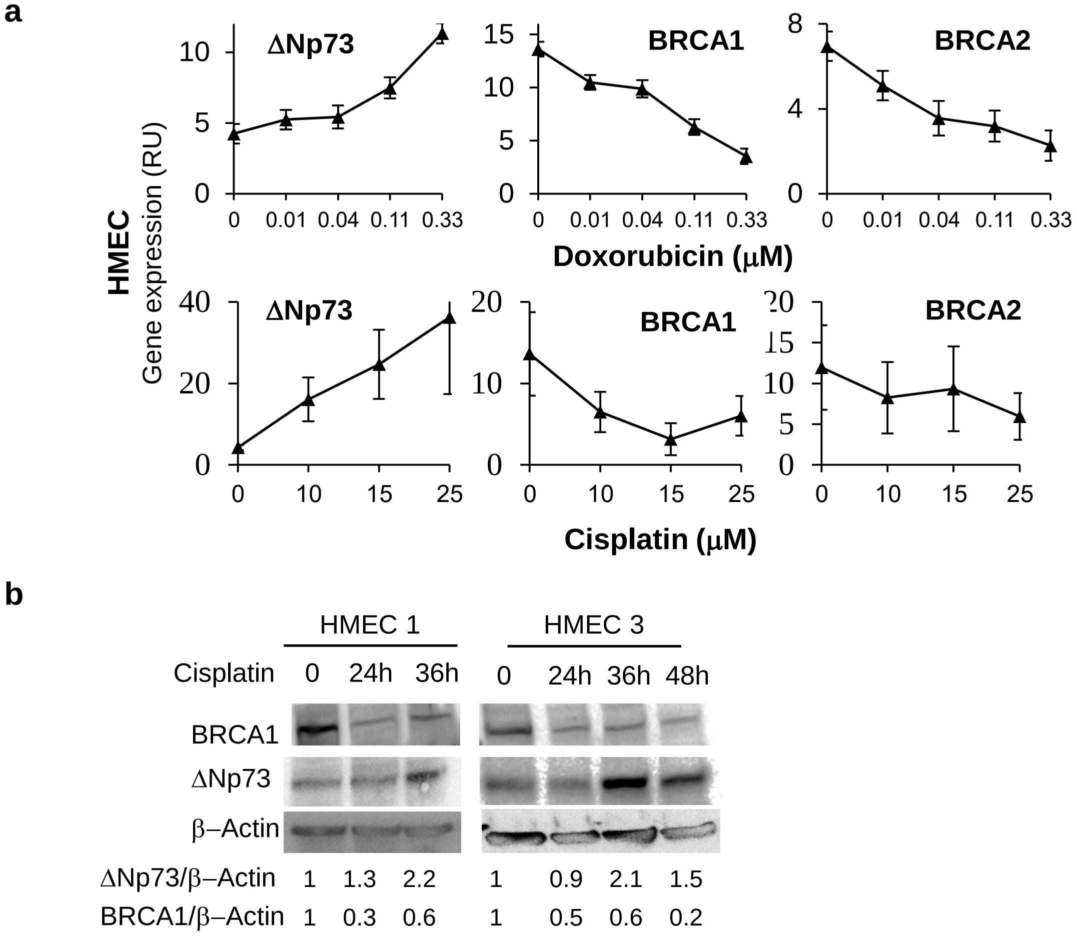

Fig 6. Expression of BRCA1 and deltaNp73 mRNA and protein in HMECs in response to cisplatin. a Gene expression (mRNA) of BRCA1 and BRCA2 was reduced while deltaNp73 was induced in HMECs (*BRCA*-wt) following exposure to doxorubicin or cisplatin for 24 hours. Gene expression was measured by RT-qPCR and normalized to GAPDH (RU = $2^{\Delta CT}$ x 10^4). Data points represent the average +/- S.E.M (n = HMECs from 4 individuals). **b** Western blots showing the reduction of BRCA1 and induction of deltaNp73 proteins in HMECs from 2 individuals following treatment with 20 μM cisplatin for the indicated time points.

1.12 Figure S7

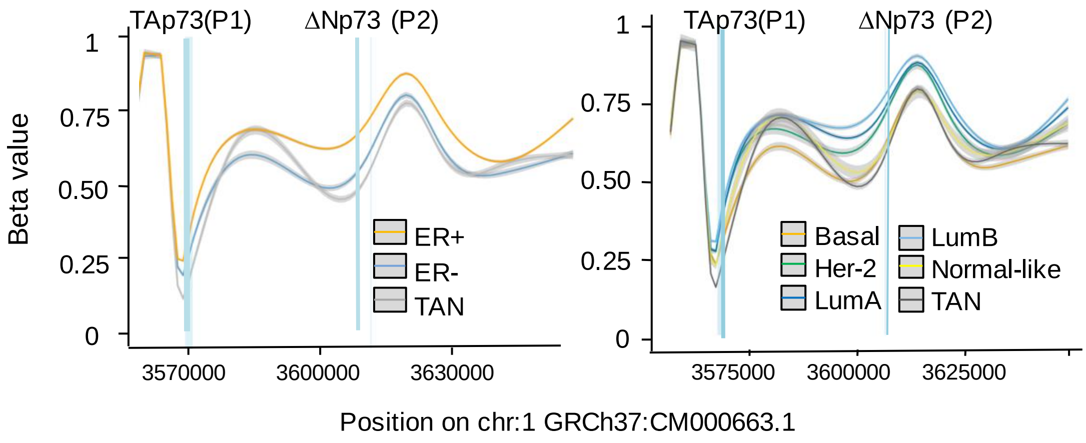

Fig. S7 Methylation of the *TP73* gene in breast tumors. TCGA analysis showing average methylation (beta values) across the entire *TP73* gene locus in breast tumors grouped according to estrogen receptor status (left) or grouped by PAM50 intrinsic tumor subtypes (right). The *TAp73* (P1) and *deltaNp73* (P2) promoter regions are marked by strips of light blue. (n= ER positive;584, ER negative;153, tumor adjacent normal tissue (TAN);97, lumA;238; LumB;267; Her2;117; basal;127, normal like;26).

1.13 Figure S8

a

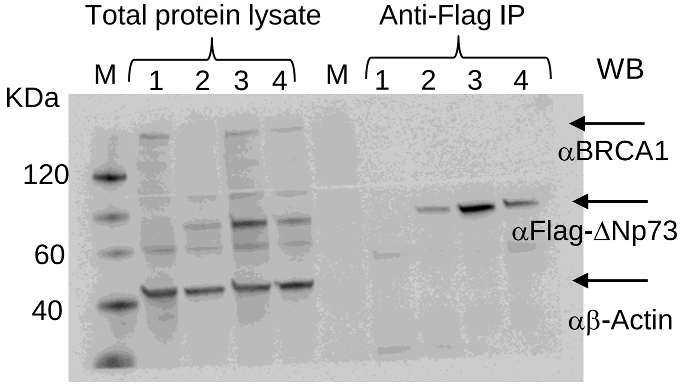

Lane #	**1**	**2**	**3**	**4**
0.1µg/ml doxycycline	-	+	+	+
0.33µM Doxorubicin	-	-	+	-
15µM Cisplatin	-	-	-	+

b

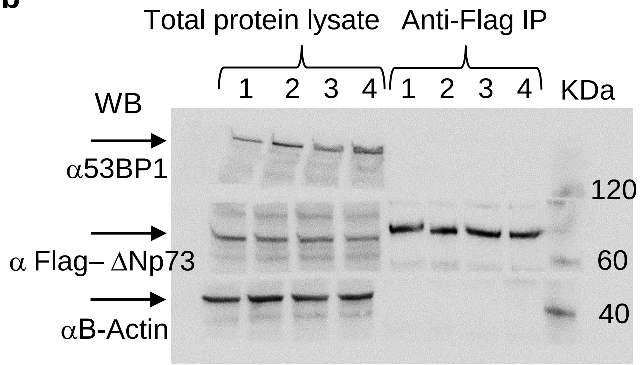

Lane #	**1**	**2**	**3**	**4**
0.1µg/ml Doxycycline	+	+	+	+
0.33µM doxorubicin	+	-	+	-
shBRCA1#1	+	+	-	-

Fig. S8 BRCA1 and 53BP1 did not co-precipitate with deltaNp73 in MCF10A.

a. MCF10A were induced by doxycycline to over-express exogenous flag-deltaNp73. Simultaneously, DNA damage was induced by doxorubicin or cisplatin for 24 hours. Cell protein lysates were either loaded directly on 2-14% gradient Acrylamide gel or following immunoprecipitation (IP) with anti-flag antibodies. Western blots (WB) were performed with antibodies to BRCA1, Flag-deltaNp73 and beta-Actin. The BRCA1 and the flag-deltaNp73 were detected in the total lysates whereas only flag-deltaNp73 was detected in the IP. **b**. A duplicate Similar experiment as described in a, where anti 53BP1antibodies detected the expected protein in the total lysate, but not in the anti-flag IPs.

Unprocessed western blot images

Breast-specific epigenetic regulation of deltaNp73 may facilitate cancer susceptibility in BRCA1-mutated HMECs

Ayelet Avraham, Susanna Feldman, Sean Soonweng Cho, Ayala Kol, Lior Heler, Emmanuela Riklin-Nahmias, Avishay Sella, Tamar Karni, Tanir M. Allweis, Saraswati Sukumar, Ella Evron.

Corresponding author: Ayelet Avraham, email: AyeletA@shamir.gov.il

General remarks

1. All blots were cut to pieces horizontally or vertically before incubation with the specific antibodies according to the expected molecular weights of the corresponding proteins.
2. Most images contain more than one blot of more than one gel that were exposed together. These are annotated.
3. The relevant lanes are marked in blue.
4. Photographs of the original blots with M.W. colored markers are presented next to the images. The relevant lanes are framed in blue.

Figure 2c

HMEC 1

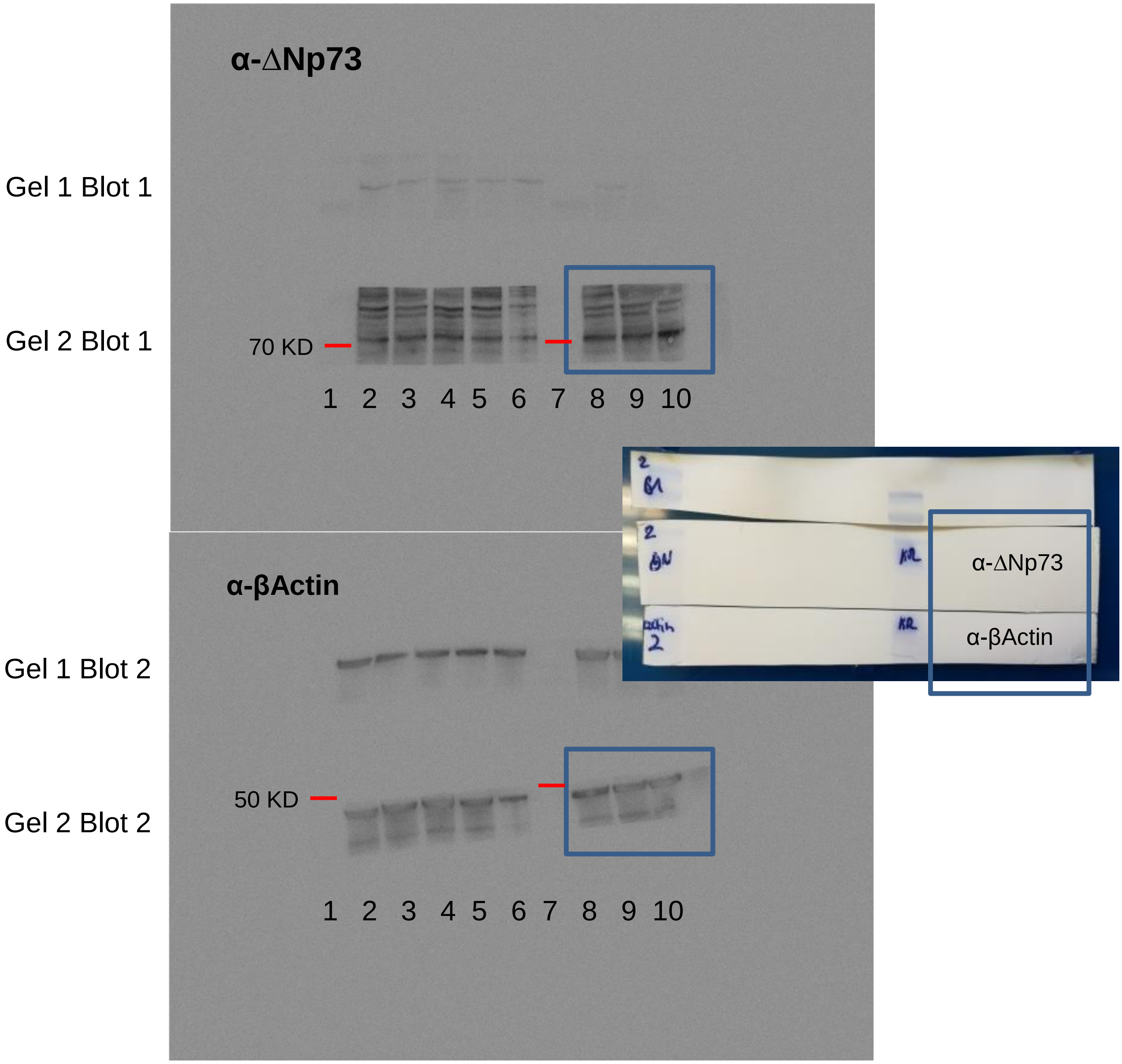

1. Marker
2. NT
3. NT 24h
4. NT 36 h
5. Doxorubicin 0.11μM 24h
6. Doxorubicin 0.33 μM 24h
7. Marker
8. NT
9. Cisplatin 20μM 24h
10. Cisplatin 20μM 36h

Figure 2c

HMEC 2

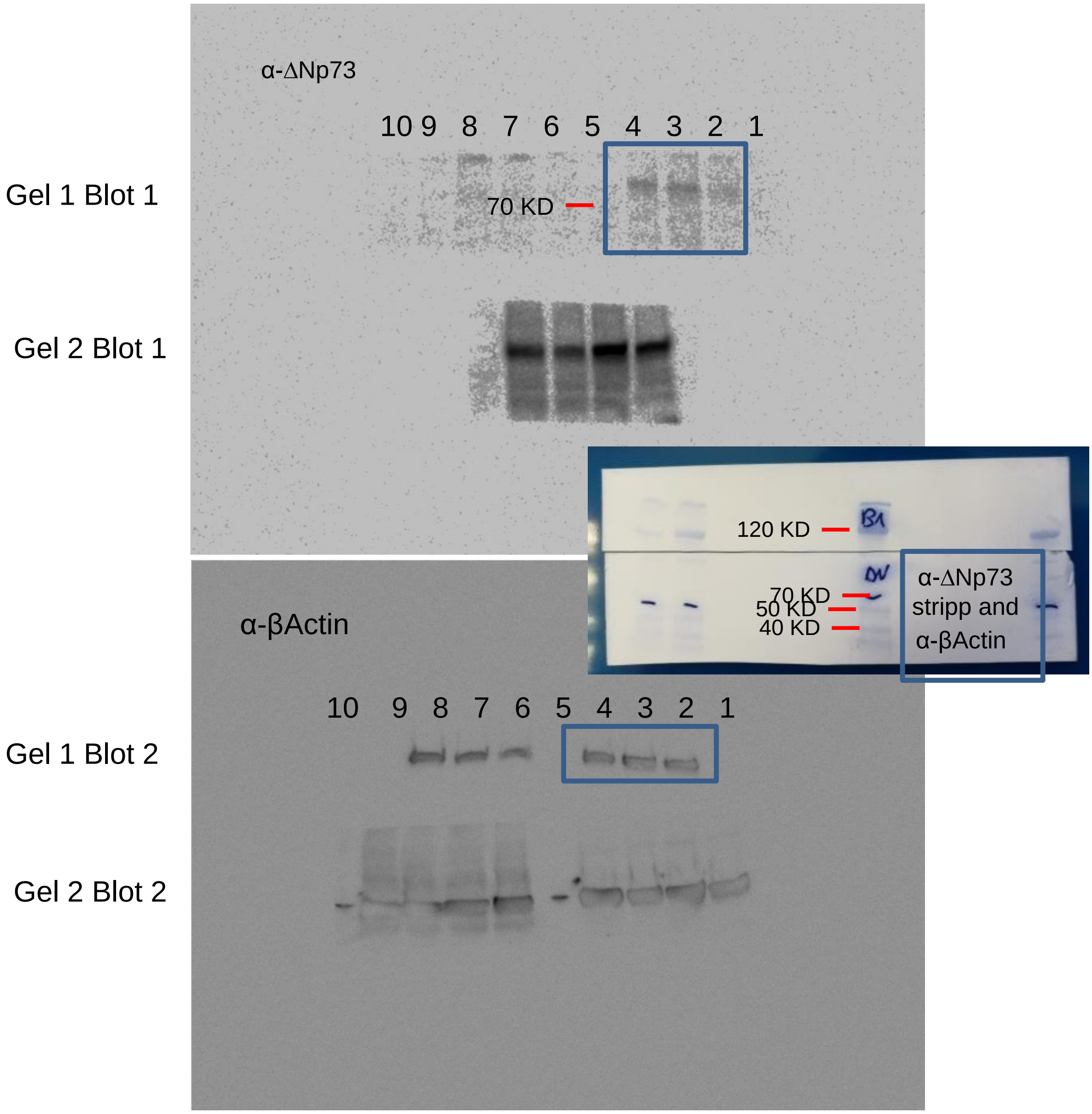

1. Marker
2. NT
3. Cisplatin 20µM 36h
4. Cisplatin 20µM 48h
5. Marker
6. NT
7. Doxorubicin 0.33µM 24h
8. Doxorubicin 0.66 µM 24h
9. Marker
10. Marker

Figure 2c

HMEC 3

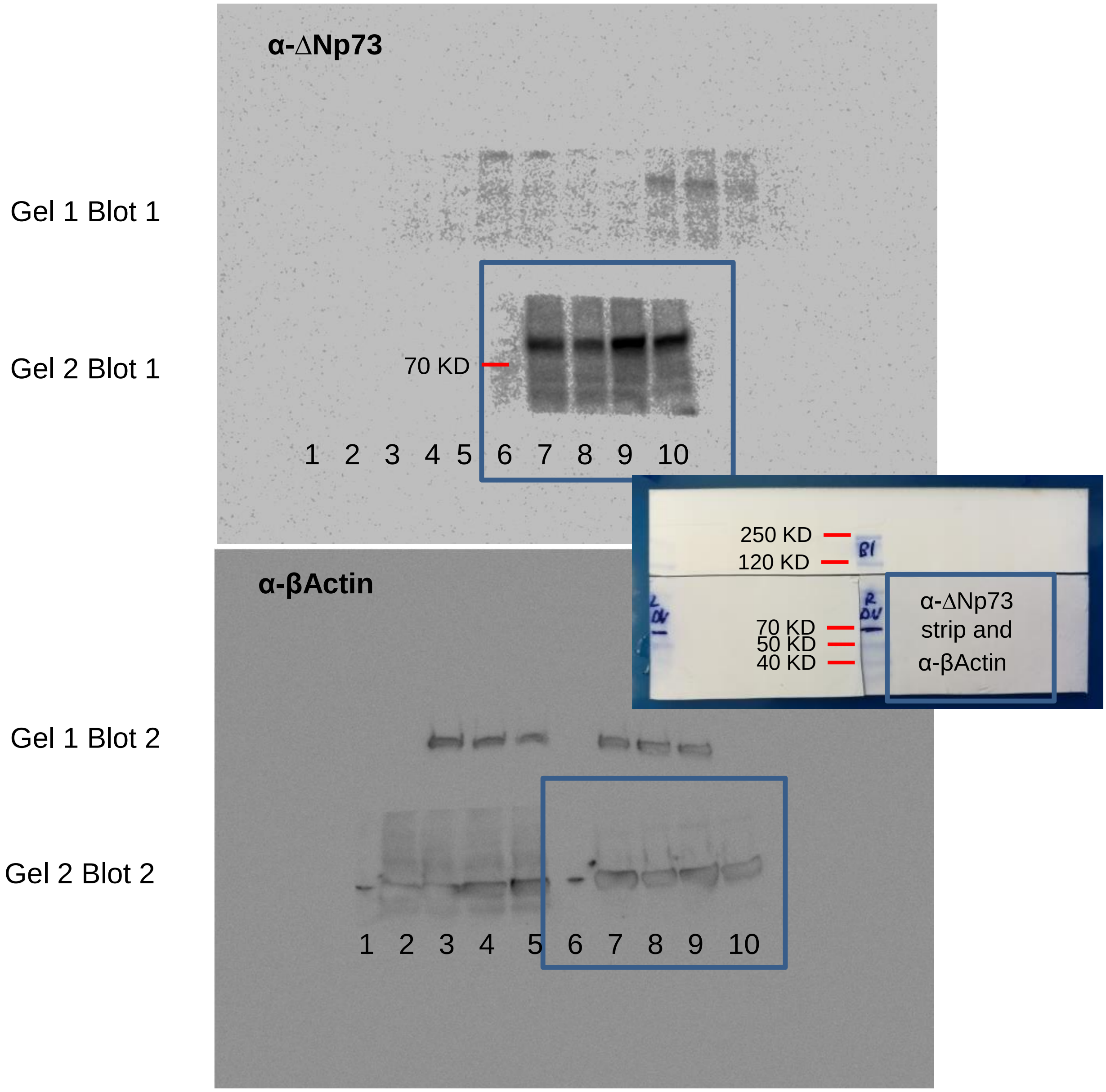

1. Marker
2. IR sample
3. IR sample
4. IR sample
5. IR sample
6. Marker
7. NT
8. Cisplatin 20µM 24h
9. Cisplatin 20µM 36h
10. Cisplatin 20µM 48h

(1–6:) Samples of another experiment

Figure 4c

MCF10A /shBRCA1#1

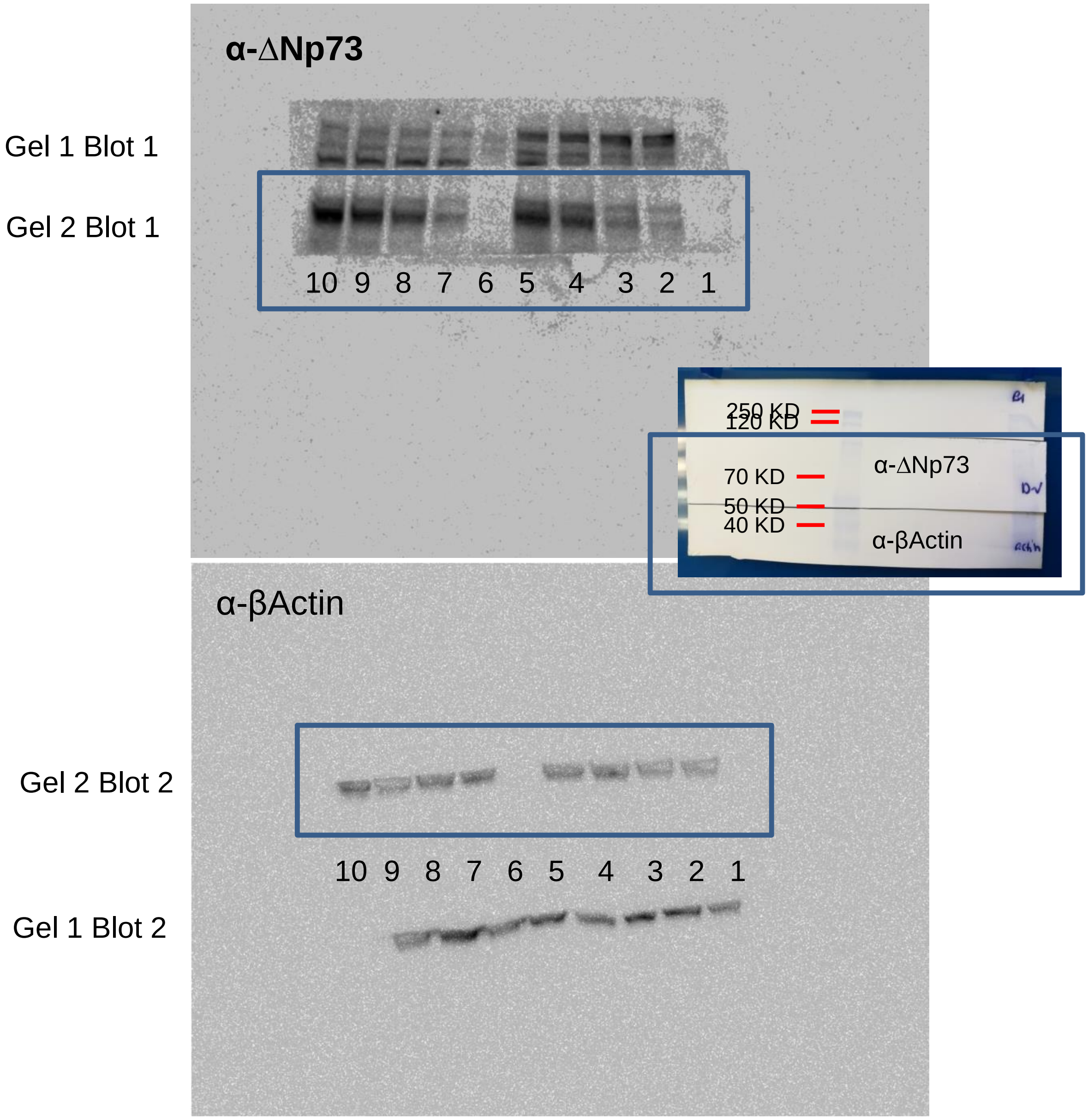

1. Marker
2. MCF10A NT
3. MACF10A Cisplatin 5µM
4. MACF10A Cisplatin 10µM
5. MCF10A Cisplatin 15µM
6. Marker
7. MCF10A /shBRCA1#1 NT
8. MACF10A /shBRCA1#1 Cisplatin 5µM
9. MACF10A /shBRCA1#1 Cisplatin 10µM
10. MCF10A /shBRCA1#1 Cisplatin 15µM

Figure 4c

MCF10A /shBRCA1#2

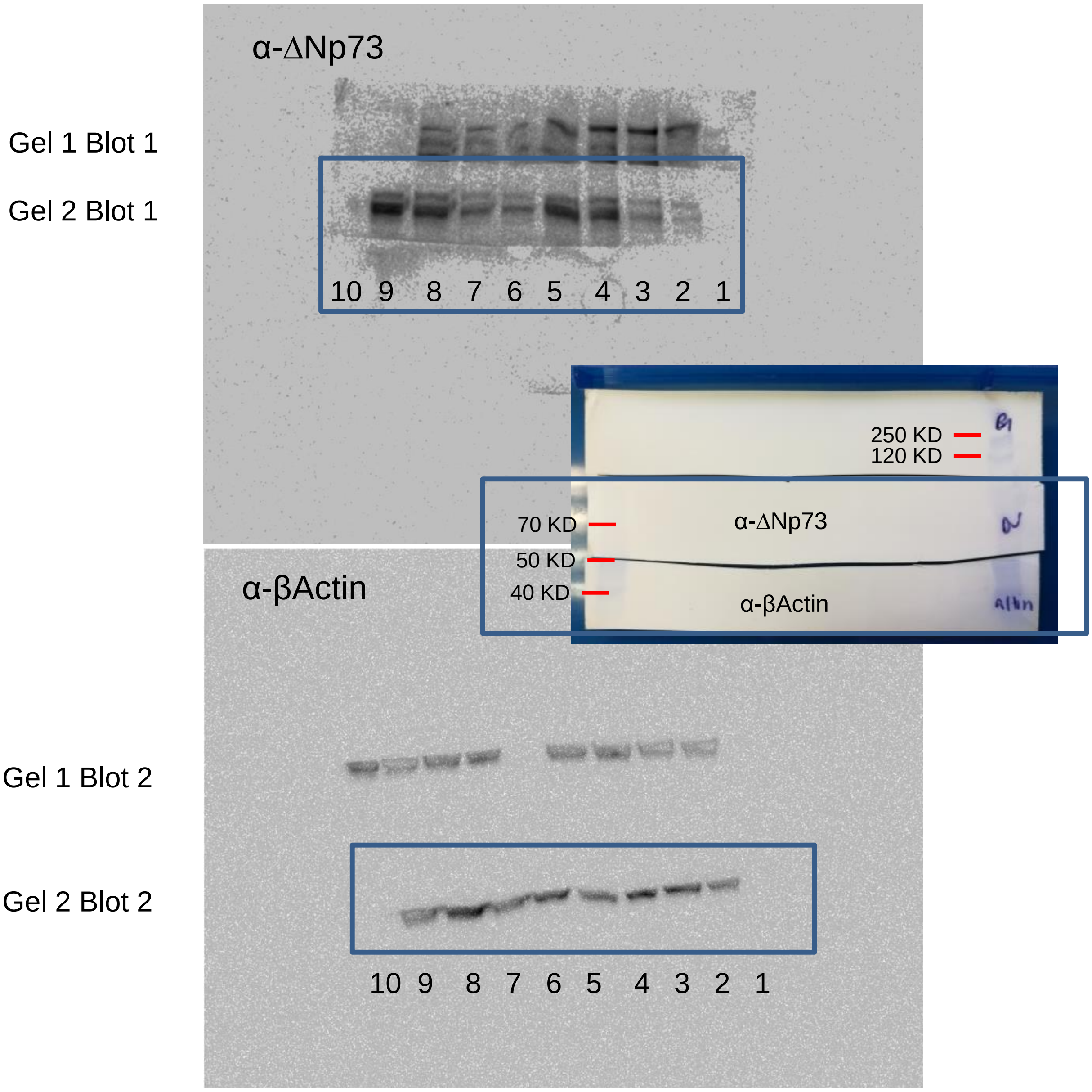

1. Marker
2. MCF10A NT
3. MACF10A Cisplatin 5µM
4. MACF10A Cisplatin 10µM
5. MCF10A Cisplatin 15µM
6. MCF10A /shBRCA1#2 NT
7. MACF10A /shBRCA1#2 Cisplatin 5µM
8. MACF10A /shBRCA1#2 Cisplatin 10µM
9. MCF10A /shBRCA1#2 Cisplatin 15µM
10. Marker

Figure 5a

MCF10A iΔNp73

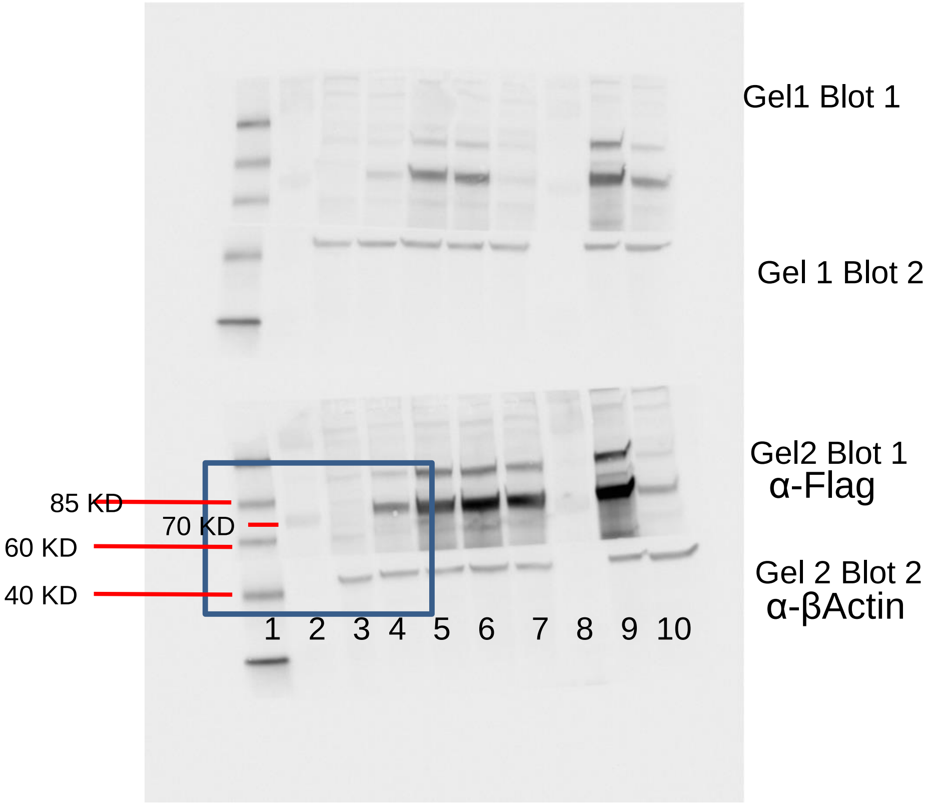

1. Marker Fluorescent
2. Marker
3. MCF10A iΔNp73 NT
4. MCF10A iΔNp73 +Doxycycline 0.1μg/ml 24h
5. MCF10A iΔNp73 +Doxycycline 0.1μg/ml 48h
6. MCF10A iΔNp73 +Doxycycline 1μg/ml 24h
7. MCF10A iΔNp73 +Doxycycline 1μg/ml 48h
8. Marker
9. MCF10A iΔNp73 +Doxycycline 1μg/ml 24h
10. MCF10A iΔNp73 +Doxycycline 0.1μg/ml 24h

Figure 5a

MCF10A iΔNp73 shBRCA1#1
MCF10A iΔNp73 shBRCA1#2

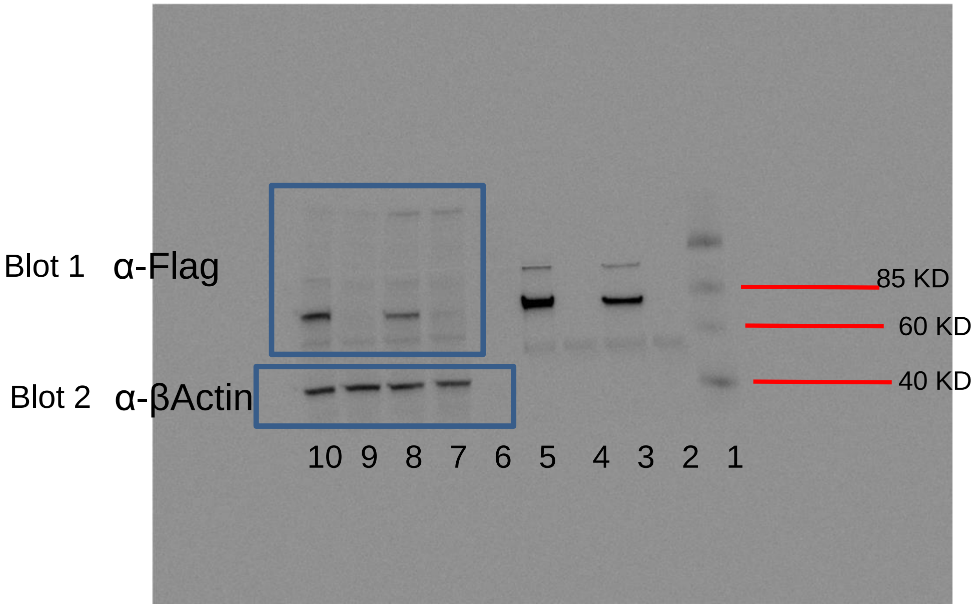

1. Marker Fluorescent
2. MCF10A iΔNp73 shBRCA1#1 (IP)
3. MCF10A iΔNp73 shBRCA1#1 +Doxycycline 0.01μg/ml (IP)
4. MCF10A iΔNp73 shBRCA1#2 (IP)
5. MCF10A iΔNp73 shBRCA1#2 +Doxycycline 0.01μg/ml (IP)
6. Marker
7. **MCF10A iΔNp73 shBRCA1#1**
8. **MCF10A iΔNp73 shBRCA1#1 +Doxycycline 0.01μg/ml**
9. **MCF10A iΔNp73 shBRCA1#2**
10. **MCF10A iΔNp73 shBRCA1#2 +Doxycycline 0.01μg/ml**

Figure 5c

MCF10A iΔNp73

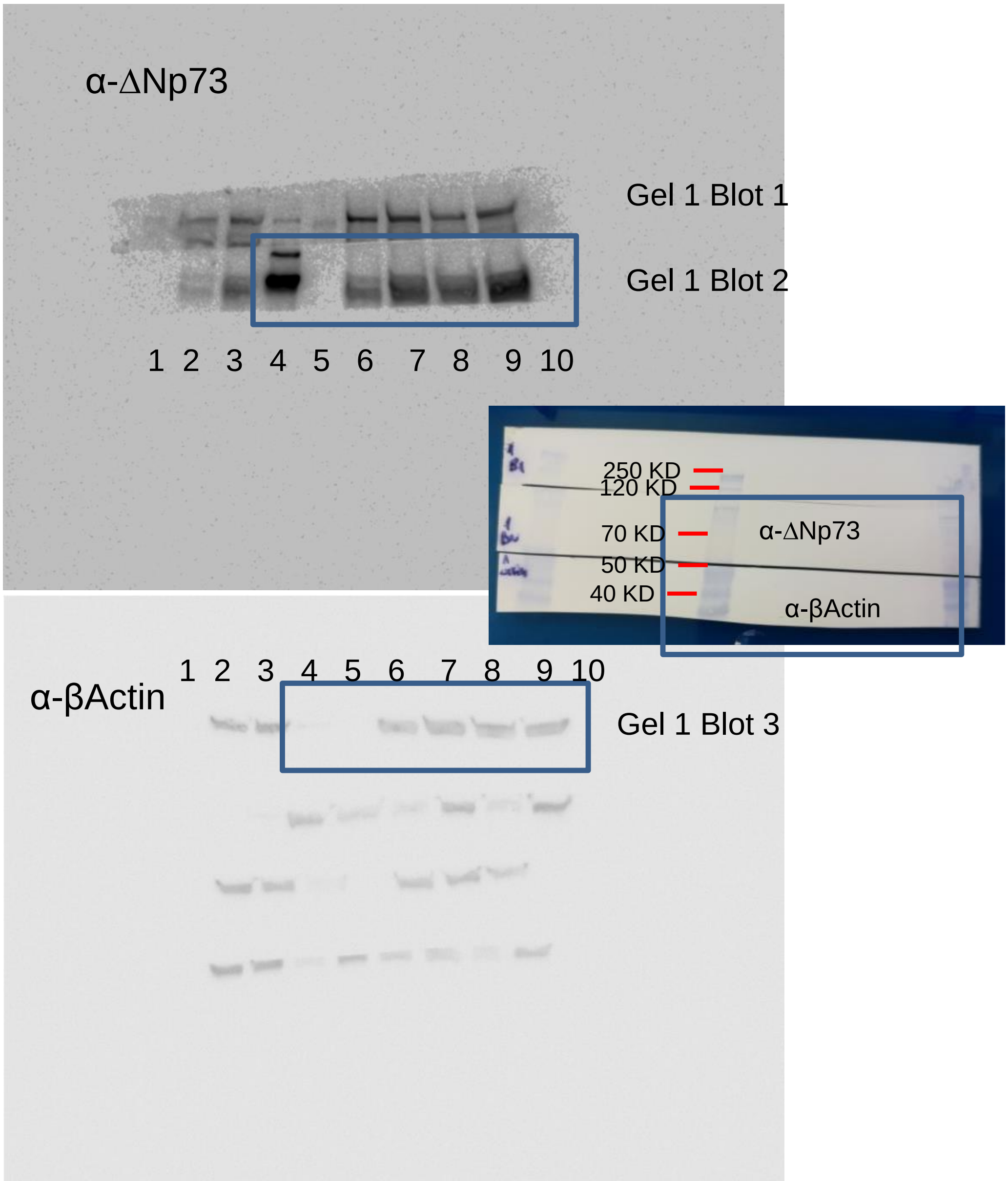

1. Marker
2. MCF10A NT
3. MCF10A Cisplatin 15μM
4. **MCF10A iΔNp73 +Doxycycline (loaded 1/20 of the amount)**
5. **Marker**
6. **MCF10A iΔNp73 NT**
7. **MCF10A iΔNp73 Cisplatin 5μM**
8. **MCF10A iΔNp73 Cisplatin 10μM**
9. **MCF10A iΔNp73 Cisplatin 15μM**
10. Marker

Figure 6c

MCF10A iΔNp73

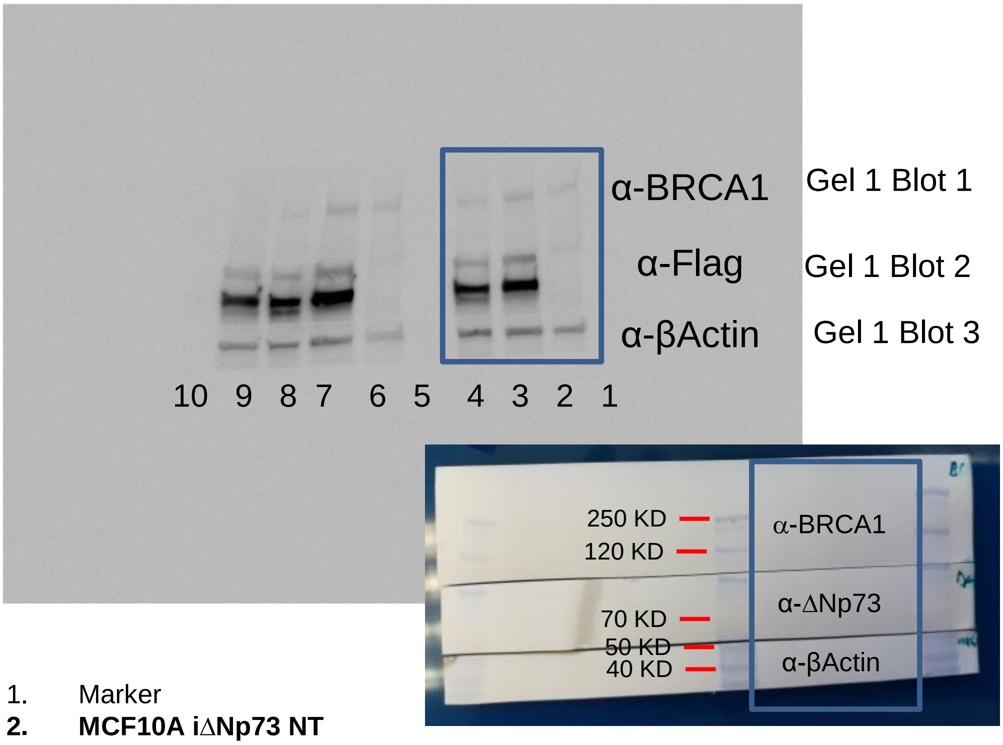

1. Marker
2. **MCF10A iΔNp73 NT**
3. **MCF10A iΔNp73 +Doxycycline 0.01μg/ml 24h**
4. **MCF10A iΔNp73 +Doxycycline 0.01μg/ml 72h**
5. Marker
6. MCF10A iΔNp73 NT
7. MCF10A iΔNp73 +Doxycycline 1μg/ml 24h
8. MCF10A iΔNp73 +Doxycycline 1μg/ml 72h
9. MCF10A iΔNp73 +Doxycycline 1μg/ml 7days
10. Marker

Figure 6c same as figure 4c

MCF10A /shBRCA1#1

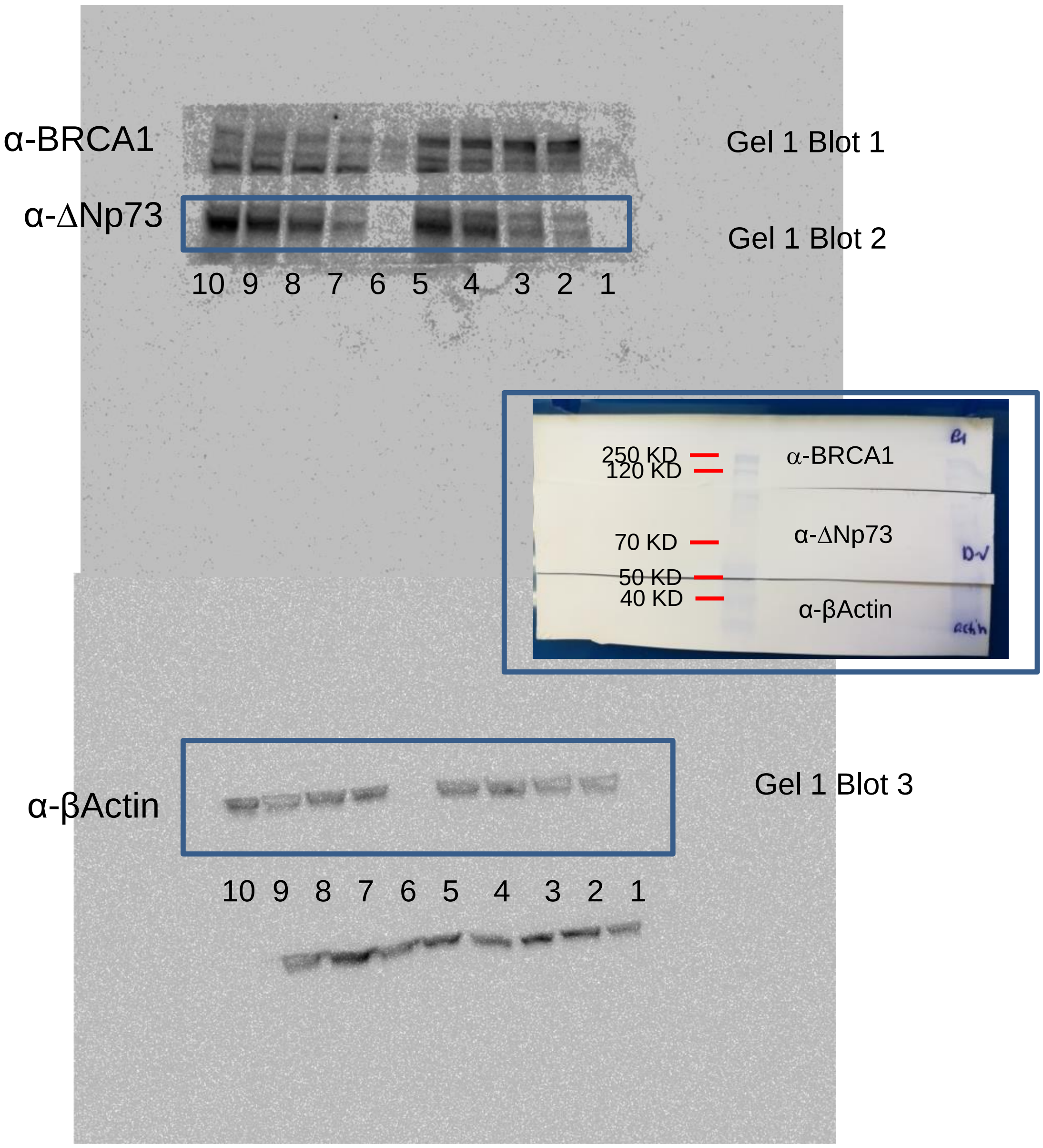

1. **Marker**
2. **MCF10A NT**
3. **MACF10A Cisplatin 5μM**
4. **MACF10A Cisplatin 10μM**
5. **MCF10A Cisplatin 15μM**
6. **Marker**
7. **MCF10A /shBRCA1#1 NT**
8. **MACF10A /shBRCA1#1 Cisplatin 5μM**
9. **MACF10A /shBRCA1#1 Cisplatin 10μM**
10. **MCF10A /shBRCA1#1 Cisplatin 15μM**

Figure S5

MCF10A /shBRCA1#1 /shBRCA1#2

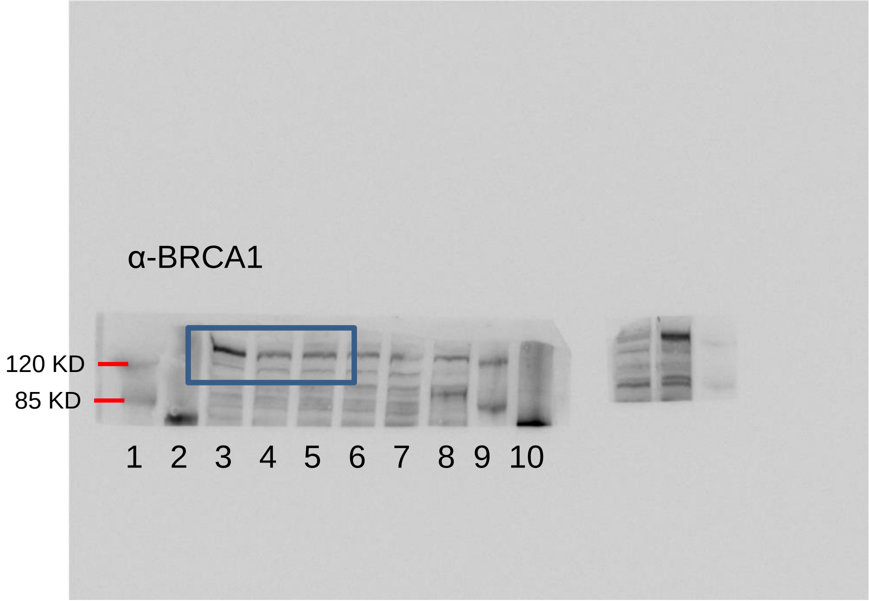

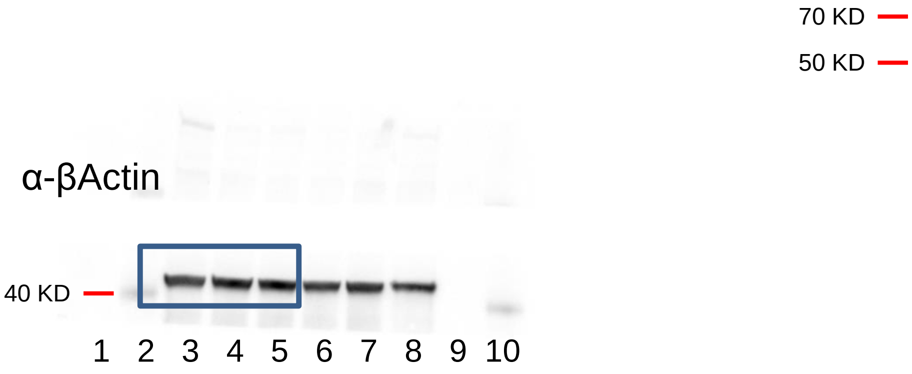

1. Marker Fluorescent
2. Marker
3. **MCF10A**
4. **MCF10A shBRCA1#1**
5. **MCF10A shBRCA1#2**
6. MCF10A shBRCA1#3
7. MCF10A shBRCA1#1+2+3
8. MCF10A shBRCA1#1+2+3
9. Marker Fluorescent
10. Marker

Figure S5

MCF10A iΔNp73 /shBRCA1#1 shBRCA1#2

Gel 1 Blot 1

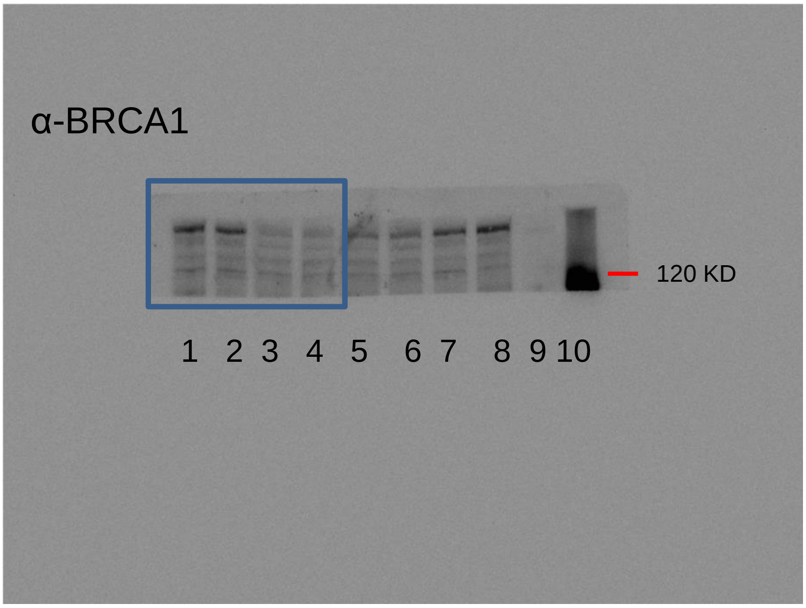

Gel 1 Blot 2

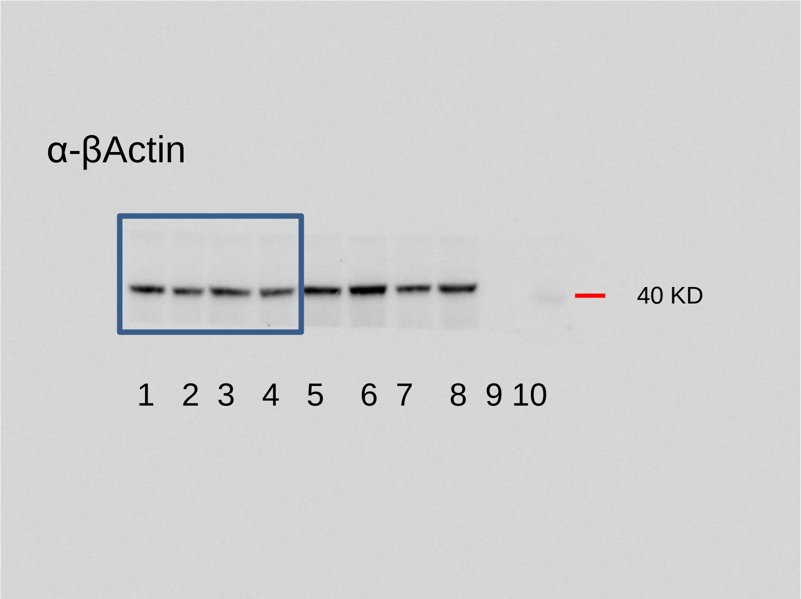

1. **MCF10A**
2. **MCF10A iΔNp73**
3. **MCF10A iΔNp73 shBRCA1#1**
4. **MCF10A iΔNp73 shBRCA1#2**
5. MCF10A iΔNp73 shBRCA1#3
6. MCF10A iΔNp73 shBRCA1#1+2+3
7. MCF10A iΔNp73
8. MCF10A
9. Marker
10. Marker Fluorescent

Figure 6S

HMEC 1

Gel 1 Blot 1

Gel 1 Blot 2

1. Marker
2. NT
3. Doxorubicin 0.66μM 24h
4. Doxorubicin 1μM 24h
5. Cisplatin 10μM 24h
6. Cisplatin 15μM 24h
7. Marker
8. **NT**
9. **Cisplatin 20μM 24h**
10. **Cisplatin 20μM 36h**

Gel 1 Blot 3

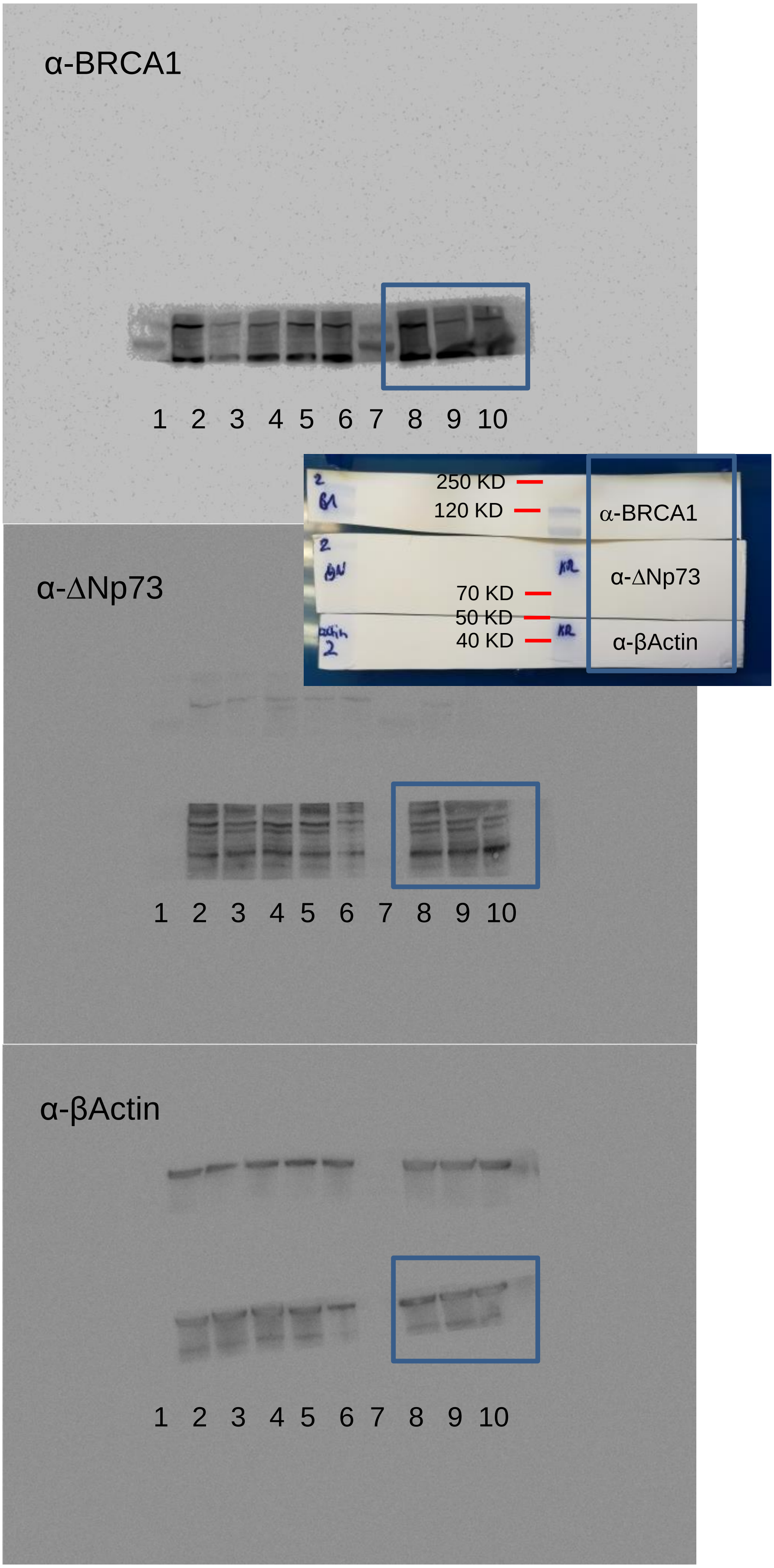

Figure 6S

HMEC 3

Gel 2 Blot 1

1. Marker
2. NT
3. Cisplatin 20μM
4. Cisplatin 20μM
5. Cisplatin 20μM 48h
6. Marker
7. **NT**
8. **Cisplatin 20μM 24h**
9. **Cisplatin 20μM 36h**
10. **Cisplatin 20μM 48h**

Gel 2 Blot 2

Gel 2 Blot 3

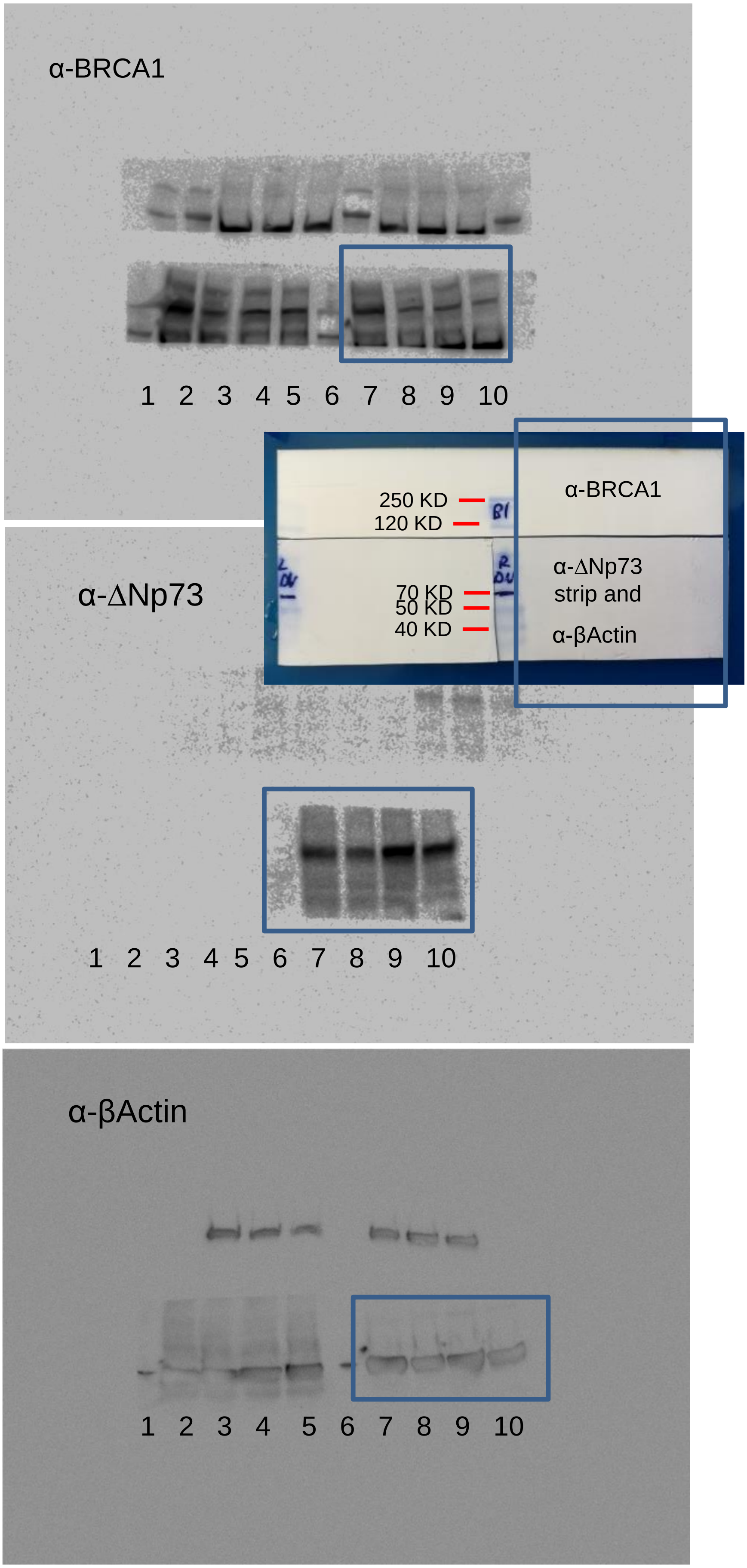

Figure S8a

MCF10A iΔNp73

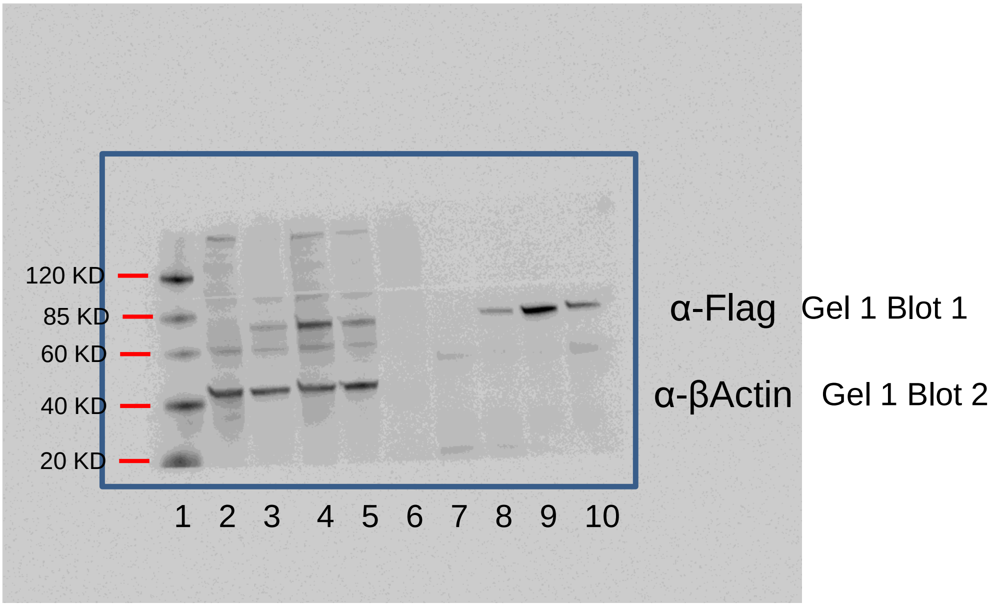

1. **Marker Fluorescent**
2. **MCF10A iΔNp73 NT**
3. **MCF10A iΔNp73 +Doxycycline 0.1μg/ml**
4. **MCF10A iΔNp73 +Doxycycline 0.1μg/ml +Doxorubicin 0.33μM**
5. **MCF10A iΔNp73 +Doxycycline 0.1μg/ml +Cisplatin 15μM**
6. **Marker**
7. **MCF10A iΔNp73 NT (IP)**
8. **MCF10A iΔNp73 +Doxycycline 0.1μg/ml (IP)**
9. **MCF10A iΔNp73 +Doxycycline 0.1μg/ml +Doxorubicin 0.33μM(IP)**
10. **MCF10A iΔNp73 +Doxycycline 0.1μg/ml +Cisplatin 15μM (IP)**

Figure S8b

MCF10A iΔNp73

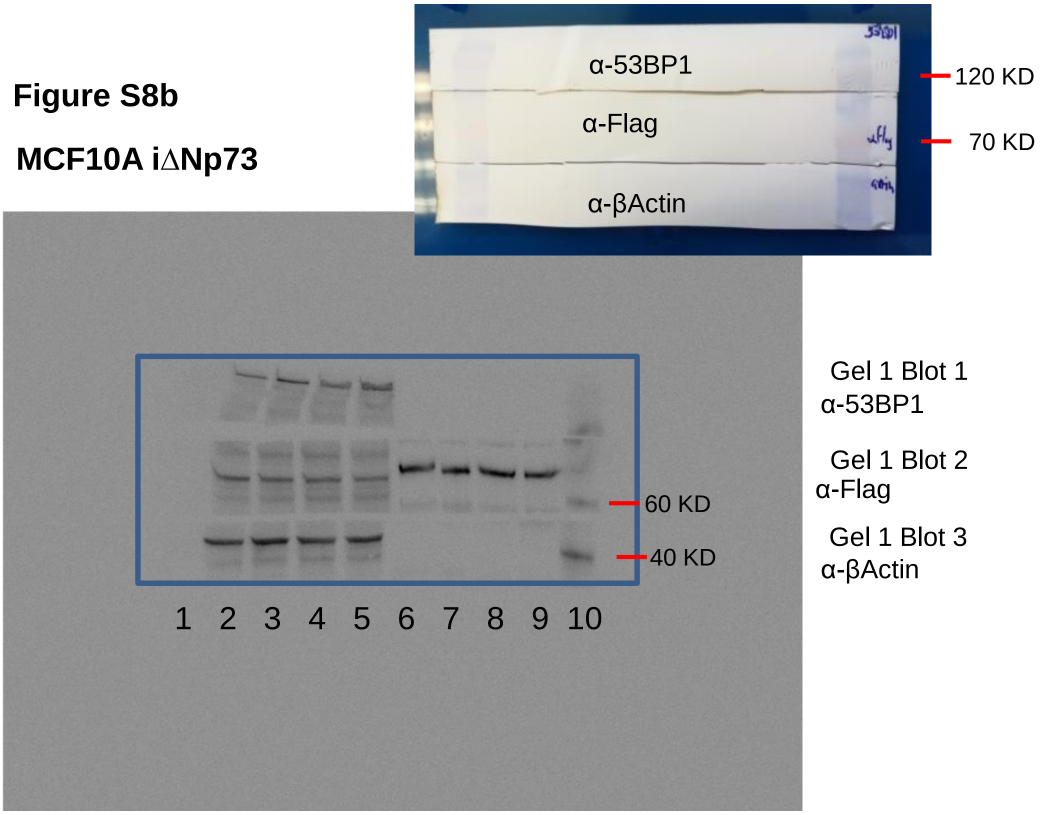

1. **Marker**
2. **MCF10A iΔNp73 /shBRCA1#1 +Doxycycline 0.1μg/ml +Doxorubicin 0.33μM**
3. **MCF10A iΔNp73 /shBRCA1#1 +Doxycycline 0.1μg/ml**
4. **MCF10A iΔNp73 +Doxycycline 0.1μg/ml +Doxorubicin 0.33μM**
5. **MCF10A iΔNp73 +Doxycycline 0.1μg/ml**
6. **MCF10A iΔNp73 /shBRCA1#1 +Doxycycline 0.1μg/ml +Doxorubicin 0.33μM (IP)**
7. **MCF10A iΔNp73 /shBRCA1#1 +Doxycycline 0.1μg/ml (IP)**
8. **MCF10A iΔNp73 +Doxycycline 0.1μg/ml +Doxorubicin 0.33μM (IP)**
9. **MCF10A iΔNp73 +Doxycycline 0.1μg/ml (IP)**
10. **Marker+ Marker Fluorescent**

Supplementary Methods

2.1

Quantitative Methylation-Specific PCR (Q-MSP)

Purification of genomic DNA from fresh tissues or cells used standard methodologies of TNES-Proteinase K and phenol-chloroform extraction followed by ethanol precipitation. Purification of DNA from FFPE Tissues used QIAamp DNA isolation Kit (Qiagen, Germantown, MD USA) according to kit instructions. DNA quantification and purity analysis used NanoDrop 2000 spectrophotometer (ThermoFisher). 0.1-1 mg of purified genomic DNA was treated with sodium bisulfite using the EZ methylation kit (Zymo research, Orange, CA, USA). Two sets of specific primers, for the un-methylated and methylated DNA forms were designed for each of the TP73 gene promoters. Primer sequences are provided below. The PCR mix contained 5 ng of sodium bisulfite-treated DNA, 200 nM of each primer and SYBR Premix Ex Taq (Takara Bio Inc, Shiga, Japan) in 10 μl final reaction volume. PCR amplification included cycles of 1 second at 95°C followed by 30 seconds at 60°C (Rotor Gene, Corbett, Australia). Human sperm DNA was used as non-methylated contro and DNA treated with SssI CpG methyltransferase (NEB, Ipswich, MA, USA) was used as methylated positive control. In addition, DNA from MCF10A cell line was used as both un-methylated and methylated controls. True amplification products were recognized by their complete overlap with the control melting points (T_M). The C_T (cycle of threshold) of the methylated and the unmethylated products for each promoter were used to calculate deltaCT ($\Delta CT^{(U-M)}$ = CT ummethylated – CT methylated). Methylation percentage for each gene were calculated accordingly: % methylation = 1/ (1 + 2^$\Delta CT^{(U-M)}$)*100.

2.2

List of Illumina methylation array (Methyl-27K) probes of the TP73 gene promoters (P1 & P2)

Illumina Probe	Illumina Probe Sequence (gene strand +)	CpG Island location*
TAp73(P1)		
cg005656884	TGTTTGGAAGGAGCTCGCGGGCGTCGGTGCGCTTGGAGTTGTTCTCTGCG(-)	1:3556965-3559547
cg043911115	CGGGCCCCATAAGCATCAGACCATAAGCAGCGCCGCCACTGAGAGCCGCT	1:3556965-3559547
cg059245836	GAAGCTGAGGCCTCGGGATTGGCACAGCCCTGCAGGTCGGAGGGAGCGCG (-)	1:3556965-3559547
cg167417103	CGCCAAGTCCCAGGGGCCGATCCAACTCCGAGGGAGCCCCTGTGTCGCCT (-)	1:3556965-3559547
cg197831141	GGCGGGGAGGCAGGGCGGGCTGCCCGGCCCCTAGGCGGGTTATATGGGCG (-)	1:3556965-3559547
cg258851082	CGGCCCATCTTTCCTGACACCCGGGTCTCTCCTGGCCGCCGCCACTAGCG (-)	1:3556965-3559547
DeltaNp73(P2)		
cg038467673	CGGCCCGCATGTTCCCCAGCATCCTCGGCTCCTGCCTCACTAGCTGCGGA(+)	1:3596889-3597535
cg166070652	TCCCATCTCCCTTAGTTCTGTCAACTGGCTGAATCCAACAACAAAACCCG(-)	1:3596889-3597535
cg251154601	CGCCGGTCAGCGCCGGCTCCATAATTAAACCCACCAGGGCTCCTTCCGAG(-)	1:3596889-3597535
cg262089304	CGGGACACCAGTTCCCTGGCGTGTGCAGACCCCCCGGCGCCTACCATGCT(+)	1:3596889-3597535

* CpG island position on chromosome 1 by Illumina map

2.3

Primers for sodium bisulfite-QMSP (quantitative methylation spesific PCR)		
Name	Direction	Sequence
TAp73_P1 _FUM	Unmethylated;forward	GGTATTTGGGTTTGTAGTTTT
TAp73_P1_RUM	Unmethylated;reverse	ATCCCAACTCATCCCCAATCA
TAp73_P1_FM	Methylated;forward	TAGGTATTTGGGTTCGTAGTTTC
TAp73_P1 _RM	Methylated; reverse	CCAACTCGTCCCCGATCG
DeltaNp73_P2_FUM3	Unmethylated;forward	TTTTGGTGTGGTTTAATATATTATT
DeltaNp73_P2_FUM3	Unmethylated; reverse	CTCATAAATATTCATCCAAATCCA
DeltaNp73_P2_FM2	Methylated; forward	TT CGGCGTTTATTATGTTGTAC
DeltaNp73_P2_FM2	Methylated; reverse	CACATCACACCTACCGTAACG
**Primers for q-PCR**		
Name	Direction	Sequence
TAp73(V1) ex1-2_GEXF	Forward	ACG TTTGAGCACCTCTGGA
TAp73(V1) ex1-2_GEXR	Reverse	TTCCGCCCACCACCTCAT
DeltaNp73(V2)ex3-4_GEXF	Forward	CATGCTGTACGTCGGTGA
DeltaNp73(V2)ex3-4_GEXF	Reverse	CTGCTCATCTGGTCCATGGT
BRCA1_GEX_F	Forward	ACCAACATGCCCACAGATCA
BRCA1_GEX_R	Reverse	CCTGTGCCAAGGGTGAATGA
BRCA2_GEX_F	Forward	GCTCAAATCAT TCCTGGTACA
BRCA2_GEX_R	Reverse	CCATACAAAGTGATAAAGGACTT
p53 _GEX_F	Forward	CGGGTCACTGCCATGGA
p53 _GEX_R	Reverse	GGTCTGAAAATGTTTCTTGACTCA
p21 _GEX_F	Forward	TGGAGACTCTCAGGGTCGAAA
p21 _GEX_R	Reverse	GGCGTTTGGAGTGGTAGAAATC
PUMA_GEX_F	Forward	GACGACCTCAACGCACAGTA
PUMA_GEX_R	Reverse	CCATGATGAGATTGTACAGGA
NOXA _GEX_F	Forward	GCGCAAGAACGCTCAA
NOXA _GEX_R	Reverse	GTTTGGATATCAGATTCAGAAG
CK7_GEX_F	Forward	GCTCCTGAAGGCTTATTCCA
CK7_GEX_R	Reverse	TCTTGTGATTGTGGGTGGTG
GAPDH_GEX_F	Forward	TGCACCACCAACTGTTAGC
GAPDH_GEX_R	Reverse	GGCATGGACTGTGGCATGAG

2.4

Sequences of siRNA and shRNA

Name	Direction	Sequence(5' to 3')	Target
siΔNp73(1)	Sense	UGACAGAACUAAGGGAGAUGGGAAA	DeltaNp73
	Antisense	UUUCCCAUCUCCCUUAGUUCUGUCA	DeltaNp73
siΔNp73(2)	Sense	GCGCCUACCAUGCUGUACGUCGGUG	DeltaNp73
	Antisense	CACCGACGUACAGCAUGGUAGGCGC	DeltaNp73
siControl	Sense	UGAAGCAAUGAGGGAUAGGGACAAA	no target
	Antisense	UUUGUCCCUAUCCCUCAUUGCUUCA	no target
shBRCA1(1)	Sense	GAGTATGCAAACAGCTATAAT	BRCA1
shBRCA1(2)	Sense	TTGCAACCTGAGGTCTATAAA	BRCA1

SiRNA was designed by BLOCK-iT™ RNAi Designer software engine (http://rnaidesigner.thermofisher.com)

Design referred to Homo sapiens tumor protein p73 (TP73), transcript variant 2, mRNA
NCBI Reference Sequence: NM_001126240.2
GGATTCAGCCAGTTGACAGAACTAAGGGAGATGGGAAAAGCGAAAATGCCAACAAACGGCCCGCATGTTCCCC
AGCATCCTCGGCTCCTGCCTCACTAGCTGCGGAGCCTCTCCCGCTCGGTCCACGCTGCCGGGCGGCCACGACC
GTGACCCTTCCCCTCGGGCCGCCCAGATCCATGCCTCGTCCCACGGGACACCAGTTCCCTGGCGTGTGCAGAC
CCCCCGGCGCCTACCATGCTGTACGTCGGTGACCCCGCACGGCACCTCGCCACG

shBRCA1 was designed using Vectorbuilder software design https://en.vectorbuilder.com/design.html according to the BRCA1 mRNA sequence NM_007294.3
